# Influence of navier-slip boundary conditions, magnetic field, and porous medium on the stability of two-dimensional channel flow

**DOI:** 10.1038/s41598-026-44816-7

**Published:** 2026-03-19

**Authors:** Palaiah A. P, Nagaraj N. Katagi, Ashwini Bhat, Manjunath Shettar

**Affiliations:** Manipal Institute of Technology, Manipal Academy of Higher Education, Manipal, India

**Keywords:** Sustainable fluid dynamics, Porous media, MHD, Navier-slip boundary, Energy efficiency, Chebyshev spectral collocation method, Engineering, Mathematics and computing, Physics

## Abstract

This study examines the linear stability of two-dimensional incompressible viscous flow through a parallel channel embedded in a porous medium under the influence of a transverse magnetic field and various slip boundary conditions. Employing the Brinkman model, the analysis integrates Navier-slip boundary formulations, including general, symmetric, and asymmetric configurations, to capture realistic surface-fluid interactions relevant to modern sustainable technologies. The modified Orr-Sommerfeld equation is solved using the Chebyshev spectral collocation method to determine velocity distributions, eigenvalues, and critical flow parameters with high numerical precision. The findings reveal that wall slip, particularly under symmetric conditions, tends to destabilise the flow by reducing wall shear stress, whereas porous resistance and asymmetric slip enhance hydrodynamic stability. An approximate 20-30% decrease in wall shear stress is related to the effect of increasing wall slope. However, the presence of a transverse magnetic field partially reduces this decrease, resulting in a 10-15% increase in wall shear stress due to magnetic damping. The role of magnetic influence and porous drag is systematically explored, offering valuable insights into optimising flow control for efficient resource utilisation, energy conservation, and environmentally responsive fluid systems. Overall, this work advances sustainable and resilient design in magnetohydrodynamic (MHD) energy systems, microfluidic devices, and porous transport technologies. Maintaining flow stability supports cleaner industrial operations and the development of energy-efficient technologies.

## Introduction

The transition from laminar to turbulent flow remains a fundamental problem in fluid dynamics due to its impact on drag, heat transfer, and flow control in engineering applications. The classical no-slip boundary condition presupposes that the fluid entirely clings to the solid surface, which has been the foundation of conventional investigations of wall-bounded flows. However, this assumption may not always be accurate, especially at micro- and nanoscales or when manufactured textures or superhydrophobic surfaces are present, as demonstrated by developments in materials science and microfabrication. As more accurate representations of fluid-wall interactions, these advancements have stimulated extensive research on slip boundary conditions.

Navier first introduced the concept of slip boundary conditions in 1823^1^. He proposed that the tangential velocity at the boundary is directly related to the shear rate at that boundary. Mathematically, the slip condition can be expressed as follows:

1 $$\begin{array}{c}{u}_{s}=k{\left.\frac{\partial u}{\partial y}\right|}_{y=0}\end{array}$$

Where $k$ denotes the slip length, ${u}_{s}$ signifies the slip velocity at the wall, and $y$ represents the wall-normal coordinate. In the scenario where $k=0$, the model aligns with the traditional no-slip condition ($u=0$), $k\to \infty$ indicates a completely slipping wall. Recent studies on superhydrophobic surfaces have indicated slip lengths in the micrometre range.

In the present study, we generalize this condition by considering different slip lengths ${k}_{1}$ and ${k}_{2}$ on the two-channel walls, which leads to

2a $$\begin{array}{c}{u-k}_{1}\frac{du}{dy}=0,v=0\,on\,y=-1\end{array}$$

2b $$\begin{array}{c}{u+k}_{2}\frac{du}{dy}=0,v=0\,on\,y=+1\end{array}$$

The streamwise and wall-normal velocity components are $u$ and $v$, respectively.

This formulation enables the modelling of coated, patterned, or asymmetrically treated channel walls commonly encountered in microfluidic and industrial applications.

Accordingly, the classical Navier slip model relates the slip velocity at the wall to the local shear rate, with the proportionality constant, the slip length, defined. Experimental studies have reported slip lengths of the order of micrometres for flows over superhydrophobic and microstructured surfaces, highlighting the growing relevance of slip boundary conditions in modern fluid mechanics. The presence of wall slip alters both the mean flow characteristics and the associated stability behaviour, with important implications for drag reduction, energy efficiency, and flow control.

The role of slip boundary conditions in flow stability has therefore attracted considerable attention in the literature. Lauga and Cossu^2^ demonstrated that wall slip can stabilise pressure-driven channel flows by increasing the critical Reynolds number. Similarly, He and Wang^3^ quantified the stabilising influence of slip within the Orr-Sommerfeld framework. More recent studies have shown that anisotropic and partial slip effects may either stabilise or destabilise the flow, depending on the orientation and magnitude of the slip parameters^4–6^.

Recent investigations have further emphasised the significant impact of slip boundary conditions on flow behaviour, particularly in configurations where slip effects are pronounced. For example, Al-Zubaidi et al.^7^ analysed squeezing flow over a Riga plate while accounting for activation energy, chemical reactions, and the effects of convective and second-order slip boundary conditions. Slip phenomena in non-Newtonian fluid flows have also been extensively examined in industrial applications such as polymer calendaring, underscoring the practical importance of slip boundary conditions^8^.

The Orr-Sommerfeld framework has also been extended to hydromagnetic and porous channel flows, where magnetic damping and porous resistance are generally found to exert a stabilising influence by suppressing velocity fluctuations and enhancing viscous dissipation^9,10^. These studies indicate that increasing either the porous drag parameter or the Hartmann number raises the critical Reynolds number and delays the onset of instability.

More recently, Xu et al.^11^ investigated electro-osmotic flow in a divergent channel under realistic boundary conditions, highlighting the importance of advanced flow modelling in complex geometries with direct biomedical relevance. In this context, electro-osmotic transport in microfluidic and physiological systems, particularly those involving non-Newtonian and multiphase fluids, remains a topic of significant research interest.

Additionally, Kudenatti et al.^12^ investigated MHD power-law fluids over moving wedges, discovering dual solutions and stabilisation by magnetic fields. In a subsequent study, Kudenatti et al.^13^ examined boundary-layer flows of non-Newtonian fluids with suction and blowing, identifying stable and unstable solution branches. Badday and Harfash^14^ studied MHD porous channel flow with slip and found that stability was enhanced with increasing $H$, permeability, and slip length. However, mode interactions and eigenvalue crossings complicate the dynamics. Thomas et al.^15^ investigated slip-induced destabilisation of crossflow instabilities over rotating disks, while Karmakar and Shukla^16^ analysed Poiseuille flow in anisotropic porous layers and highlighted the combined role of anisotropy and variable permeability in shaping stability. Dabiri and Leonard^17^ revisited the no-slip assumption, demonstrating that perturbative deviations from it predict transition Reynolds numbers consistent with experimental observations.

Recent progress includes the investigation of Magnetohydrodynamic flow of non-Newtonian fluids through porous media with variable transport properties, which has also been investigated to understand the influence of magnetic and thermal effects on Poiseuille-type flows^18^. Kumar et al.^19^ analysed linear and transient stability in Darcy-Brinkman porous media with slip and demonstrated that symmetric slip enhances stability. In contrast, asymmetric slip can produce significant transient growth. Two-phase magnetohydrodynamic flows with thermal effects in inclined channel configurations have also been examined to understand heat transfer mechanisms in complex fluids^20^. The multiphase flow behaviour of non-Newtonian fluids with wall interaction effects has also been examined to understand the influence of wall properties on flow characteristics^21^. Geetha et al.^22^ investigated the stabilising role of transverse magnetic fields on modal and nonmodal disturbances. Later, Shivaraj et al.^23^ studied MHD channel flows with slip, revealing that symmetric slip consistently improves stability, whereas asymmetric slip leads to more intricate transitions. Particle-charged non-Newtonian flows in Couette-Poiseuille configurations have been examined analytically and via perturbation methods, demonstrating the value of perturbation techniques for solving complex flow problems^24^. Slip-dependent flows in non-Newtonian fluids have also been investigated in convergent channel configurations using analytical approaches^25^. Lamesse and Ibrahim^26–29^ conducted several studies on MHD nanofluid flow over paraboloid surfaces in porous media, including Casson-dusty, Carreau, Powell–Eyring, and tangent hyperbolic models. Their works showed that magnetic field generally reduces fluid velocity, while parameters such as nanoparticle volume fraction, Darcy number, radiation, and mixed convection enhance temperature and heat transfer characteristics. These studies highlight the significant role of porous medium properties, slip effects, and non-Newtonian behavior in controlling flow and thermal performance.

Despite significant progress, comprehensive linear stability analyses that simultaneously account for porous resistance, a transverse magnetic field, and asymmetric Navier slip boundary conditions in channel flows remain scarce. Most existing investigations are restricted to no-slip or symmetric slip configurations, thereby overlooking the practical importance of asymmetric slip conditions commonly encountered on coated, micro-structured, and engineered surfaces.

Motivated by recent advances in slip-dependent and magnetohydrodynamic flows, this study addresses the gap by performing a detailed linear stability analysis of magnetohydrodynamic flow in a porous channel with wall slip effects. A general Navier slip formulation is adopted, encompassing no-slip, symmetric slip, and asymmetric slip boundary conditions at the channel walls. This framework better represents real-world applications involving manufactured and coated surfaces. The combined influence of wall slip, transverse magnetic field, and porous resistance on flow stability is systematically examined within the channel domain $y\in [-\mathrm{1,1}]$. The resulting eigenvalue problem is solved using the Chebyshev spectral collocation method, ensuring high numerical accuracy and rapid convergence. In addition to a detailed physical interpretation, the study offers new insights into flow control mechanisms in porous and magnetohydrodynamic systems.

The analysis reveals that asymmetric slip and porous resistance stabilise the flow, whereas symmetric slip tends to promote instability. These findings contrast with earlier studies that treated slip effects, magnetic fields, and porous resistance independently. The results are particularly relevant to magnetohydrodynamic and microfluidic systems where surface characteristics play a critical role.

Specifically, the linear stability of two-dimensional, incompressible, viscous flow through a parallel channel filled with porous material, subjected to a transverse magnetic field, is investigated using the Brinkman model. Modal analysis is employed to assess the effects of slip length, porosity parameters, and Hartmann number on the onset of instability. Quantitative results indicate that Navier slip can reduce wall shear stress by up to 30%, while increased porous resistance and magnetic field strength enhance flow stabilisation and increase wall shear stress. Overall, this study elucidates the interplay between slip, magnetic damping, and porous resistance in governing flow stability, with direct implications for energy systems, MHD devices, microfluidic applications, filtration systems, and related engineering technologies where stability control is essential.

## Formulation

**Fig. 1:**
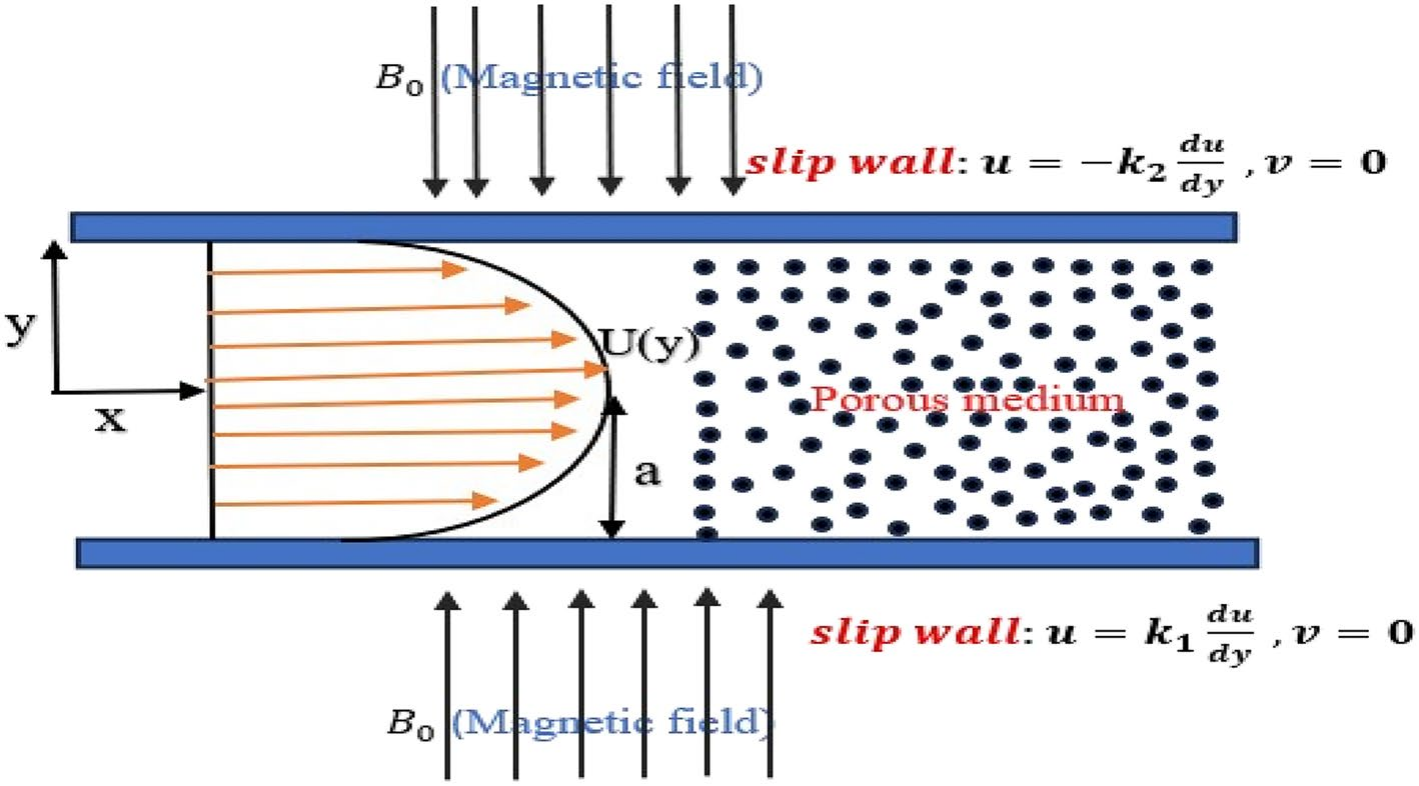
Schematic diagram of a parallel channel flow with slip conditions applied at both walls, characterized by slip lengths ${k}_{1 }\,and\,{k}_{2}$.

We analyze the two-dimensional flow of an incompressible viscous fluid through a porous medium in a parallel channel, subjected to a magnetic field ${B}_{0}$ applied along the $y$-axis, perpendicular to the channel walls, as shown in Fig. 1. The magnetic Reynolds number is assumed to be small, implying that the magnetic field induced by the fluid motion is negligible compared to the imposed field. Consequently, ${B}_{0}$ is considered uniform and steady, and the induction equation decouples from the momentum equations.

### Modal assumptions

The following assumptions govern the formulation of the mathematical model:

(i). The flow is laminar, two-dimensional, steady, and incompressible.
(ii). The fluid is Newtonian with constant physical properties.
(iii). A homogeneous porous material fills the channel, and a Darcy resistance term accounts for the porous resistance.
(iv). A uniform magnetic field ${B}_{0}$ is applied perpendicular to the channel walls in the transverse direction $(y)$.
(v). In the streamwise $(x)$ direction, the flow has fully developed.
(vi). The normal velocity at the channel walls is zero, and both channel walls are subject to Navier slip boundary conditions, which are represented by the slip lengths ${k}_{1}$ and ${k}_{2}$.

These assumptions, now widely adopted in the analysis of magnetohydrodynamic flow through porous channels, enable the essential physical effects of wall slip, magnetic damping, and porous resistance to be captured without introducing unnecessary mathematical complexity.

In two dimensions, the governing continuity and momentum equations are given by^9,30^

3 $$\begin{array}{c}\frac{\partial U}{\partial X}+\frac{\partial V}{\partial Y}=0\end{array}$$

4 $$\begin{array}{c}\rho \left(\frac{\partial U}{\partial t}+U\frac{\partial U}{\partial X}+V\frac{\partial U}{\partial Y}\right)=-\frac{\partial p}{\partial X}+\mu \left(\frac{{\partial }^{2}U}{\partial {X}^{2}}+\frac{{\partial }^{2}U}{\partial {Y}^{2}}\right)-\mu \frac{U}{K}-\sigma {B}_{0}^{2}U\end{array}$$

5 $$\begin{array}{c}\rho \left(\frac{\partial V}{\partial t}+U\frac{\partial V}{\partial X}+V\frac{\partial V}{\partial Y}\right)=-\frac{\partial p}{\partial Y}+\mu \left(\frac{{\partial }^{2}V}{\partial {X}^{2}}+\frac{{\partial }^{2}V}{\partial {Y}^{2}}\right)-\mu \frac{V}{K}\end{array}$$

Where ${\boldsymbol{U}} = (U, V)$ denotes the dimensional velocity vector, and $U\,and\,V$ are the velocity components in the *X*- and *Y*-directions, respectively, $\rho$ is the density, $\upmu$ is the dynamic viscosity, $p$ is Pressure, $\upsigma$ is the electrical conductivity, $K$ is the permeability of the porous medium.

Introducing the following dimensionless variables:

6 $$\begin{array}{c}x=\frac{X}{a},\hspace{1em}y=\frac{Y}{a},\hspace{1em}u=\frac{U}{{U}_{0}},\hspace{1em}v=\frac{V}{{U}_{0}},\hspace{1em}t=\frac{{t}^{*}{U}_{0}}{a},\hspace{1em}P=\frac{p}{\uprho {U}_{0}^{2}}\end{array}$$

Substituting equation (6) into equations (3-5), we get non-dimensional equations.

7 $$\begin{array}{c}\frac{\partial u}{\partial x}+\frac{\partial v}{\partial y}=0\end{array}$$

8 $$\begin{array}{c}\frac{\partial u}{\partial t}+u\frac{\partial u}{\partial x}+v\frac{\partial u}{\partial y}=-\frac{\partial P}{\partial x}+\frac{1}{Re}\left(\frac{{\partial }^{2}u}{\partial {x}^{2}}+\frac{{\partial }^{2}u}{\partial {y}^{2}}\right)-\left(\frac{{S}^{2}}{Re}+\frac{{H}^{2}}{Re}\right)u\end{array}$$

9 $$\begin{array}{c}\frac{\partial v}{\partial t}+u\frac{\partial v}{\partial x}+v\frac{\partial v}{\partial y}=-\frac{\partial P}{\partial y}+\frac{1}{Re}\left(\frac{{\partial }^{2}v}{\partial {x}^{2}}+\frac{{\partial }^{2}v}{\partial {y}^{2}}\right)-\frac{{S}^{2}}{Re}v\end{array}$$

Where $u = (u, v)$ denotes the dimensionless velocity vector, with $u\,and\,v$ representing the velocity components in the *x*- and *y*-directions, respectively, $a$ is the channel half-width, ${U}_{0}$ is the characteristic velocity, $\upnu =\frac{\upmu }{\uprho }$ is the kinematic viscosity, $Da$ is the Darcy number, $Re$ is the Reynolds number, $H$ is the magnetic field parameter, and $S$ is the shape factor.

$$Re=\frac{{U}_{0}a}{\upnu },\,Da=\frac{K}{{a}^{2}},\,{ S}^{2}=\frac{1}{Da},\,{ H}^{2}=\frac{\upsigma {B}_{0}^{2}{a}^{2}}{\rm{\rho \nu }{U}_{0}}$$

The flow is modelled to satisfy different slip boundary conditions on the lower and upper surfaces, with slip lengths ${k}_{1}$ and ${k}_{2}$ applied along the walls $y=\pm 1,$ respectively.

The general form of the steady, unidirectional base flow, which satisfies equations (7-9), is given by

10 $$\begin{array}{c}u=\left(\overline{u }\left(y\right),0\right)\end{array}$$

The velocity profile $\overline{u }\left(y\right)$ is obtained by solving the dimensionless governing equations with the applied slip conditions imposed on $y=\pm 1$.

## Linear stability analysis

In the stability analysis, attention is restricted to two-dimensional disturbances, for which Squire’s transformation^31^ is applicable. Under these conditions, the system’s behaviour can be more clearly examined by introducing small perturbations to the base flow.

11a $$\begin{array}{c}u\left(x,y,t\right)=\overline{u }\left(y\right)+{u}^{\prime}\left(x,y,t\right),\end{array}$$

11b $$\begin{array}{c}v\left(x,y,t\right)={v}^{\prime}\left(x,y,t\right),\end{array}$$

11c $$\begin{array}{c}P\left(x,y,t\right)=\overline{P }\left(x\right)+{P}^{\prime}\left(x,y,t\right)\end{array}$$

Where ${{u}} = (u, v)$ is the velocity vector, $\overline{{{u}} }= (\overline{u }\left(y\right), 0)$ is the base-flow velocity

vector, and ${{u}}\boldsymbol{^{\prime}} = (u^{\prime}, v^{\prime})$ represents the disturbance velocity vector.

Substituting equation (11a) into (7-9) and simplifying, we get linearized perturbation equations.

12 $$\begin{array}{c}\frac{\partial {u}^{\prime}}{\partial x}+\frac{\partial {v}^{\prime}}{\partial y}=0\end{array}$$

13 $$\begin{array}{c}\frac{\partial {u}^{\prime}}{\partial t}+\overline{u}\frac{\partial {u}^{\prime}}{\partial x}+{v}^{\prime}\frac{d\overline{u}}{dy }=-\frac{\partial {P}^{\prime}}{\partial x}+\frac{1}{Re}\left(\frac{{\partial }^{2}{u}^{\prime}}{\partial {x}^{2}}+\frac{{\partial }^{2}{u}^{\prime}}{\partial {y}^{2}}\right)-\frac{\left({S}^{2}+{H}^{2}\right)}{Re}{u}^{\prime}\end{array}$$

14 $$\begin{array}{c}\frac{\partial {v}^{\prime}}{\partial t}+\overline{u}\frac{\partial {v}^{\prime}}{\partial x}=-\frac{\partial {P}^{\prime}}{\partial y}+\frac{1}{Re}\left(\frac{{\partial }^{2}{v}^{\prime}}{\partial {x}^{2}}+\frac{{\partial }^{2}{v}^{\prime}}{\partial {y}^{2}}\right)-\frac{{S}^{2}}{Re}{v}^{\prime}\end{array}$$

To analyse the stability of the flow, we introduce small perturbations in the form of normal modes. The stream function perturbation is therefore assumed to be

15 $$\begin{array}{c}\psi \left(x,y,t\right)=\phi \left(y\right){e}^{i\rm{\alpha }\left(x-ct\right)}\end{array}$$

Where $\upphi \left(y\right)$ is the amplitude function and $\rm{\alpha}\,\&\,c$ are the disturbance wavenumber and complex wave speed, respectively.

The disturbance velocity vector is provided by ${{u}}^{\prime} = (u^{\prime}, v^{\prime})$, and the disturbance velocity components are derived from the streamfunction.

16 $$\begin{array}{c}{u}^{\prime}\left(x,y,t\right)=\frac{\partial\uppsi }{\partial y}={\upphi}^{\prime}\left(y\right){e}^{i\rm{\alpha }\left(x-ct\right)}\end{array}$$

17 $$\begin{array}{c}{v}^{\prime}\left(x,y,t\right)=-\frac{\partial\uppsi }{\partial x}=-i\alpha \phi \left(y\right){e}^{i\rm{\alpha }\left(x-ct\right)}\end{array}$$

After substituting equations (16) and (17) into equations (12-14) and simplifying, we get the modified Orr-Sommerfeld equation:

18 $$\begin{array}{c}{\left({D}^{2}-{\rm{\alpha }}^{2}\right)}^{2}\phi -i\alpha Re\left[\left(\overline{\mathrm{u} }-\mathrm{c}\right)\left({\mathrm{D}}^{2}-{\rm{\alpha }}^{2}\right)\upphi -{\overline{\mathrm{u}} }^{\rm{^{\prime}}\rm{^{\prime}}}\upphi \right]-\left({S}^{2}+{H}^{2}\right){D}^{2}+{\rm{\alpha }}^{2}{S}^{2}\phi =0\end{array}$$

Where $D$ stands for differentiation with respect to the wall-normal coordinate $y$, and $\upphi$ represents the scalar amplitude of the streamfunction disturbance.

When the porous and magnetic effects are neglected (S = H = 0), equation (18) reduces to the classical Orr-Sommerfeld equation^32,33^:

$$\begin{array}{c}{\left({D}^{2}-{\rm{\alpha }}^{2}\right)}^{2}\phi =i\alpha Re\left[\left(\overline{\mathrm{u} }-\mathrm{c}\right)\left({\mathrm{D}}^{2}-{\rm{\alpha }}^{2}\right)\upphi -{\overline{\mathrm{u}} }^{\rm{^{\prime}}\rm{^{\prime}}}\upphi \right]\end{array}$$

Where ${D}^{2}$ denotes the second derivative with respect to $y, \rm{\alpha }$ is the real-valued streamwise wavenumber, $c$ is the complex phase speed (eigenvalue).

The complex wave speed *c* is expressed as

19 $$\begin{array}{c}c={c}_{r}+i{c}_{i}\end{array}$$

Where the real part ${c}_{r}$ denotes the wave phase speed, while the imaginary part ${c}_{i}$ represents the temporal growth $({c}_{i}> 0)$ or decay $({c}_{i}< 0)$ of the disturbance.

For a temporal instability analysis, the flow is said to be:

- Stable if $${c}_{i}<0,$$
- Unstable if $${c}_{i}>0$$,
- Neutral if $${c}_{i}=0$$.

The linear stability of viscous fluid flow in a porous medium within a parallel channel under a transverse magnetic field is analysed using the modified Orr-Sommerfeld equation. This equation is discretised using the Chebyshev spectral collocation method, which employs Chebyshev differentiation matrices at Gauss-Lobatto collocation points to achieve an accurate approximation of wall-normal derivatives. A total of $N = 120$ Chebyshev points are employed over the domain $y\in \left[-\mathrm{1,1}\right],$ leading to the formulation of a generalised eigenvalue problem. The resulting eigenvalue problem is solved numerically using *MATLAB R2024b* to investigate the impact of various wall boundary conditions on flow stability.

The framework accommodates classical no-slip boundaries, symmetric slip (where equal slip lengths are applied to both walls), asymmetric slip (where slip is applied only to one wall), and general Navier-slip (where different slip lengths are applied to each boundary). For each scenario, the discrete system is solved to obtain complex eigenvalues, enabling the analysis of growth rates, phase speeds, and critical Reynolds numbers. This comprehensive spectral method allows a systematic comparison of how different wall-slip conditions affect the onset of instability in magnetohydrodynamic (MHD) flow through a parallel channel filled with a porous medium.

## Boundary conditions

To examine the effect of wall slip on flow stability, four types of boundary conditions are considered: general Navier slip, no-slip, symmetric slip, and asymmetric slip. Each of these conditions alters both the base velocity profile and the associated boundary conditions required for linear stability analysis. Since they directly affect near-wall dynamics, the choice of boundary conditions plays a critical role in determining the flow’s stability behaviour.

In this context, the velocity vector is defined as $\mathrm{u}=(u,v)$, where $u$ and $v$ denote the streamwise and wall-normal velocity components, respectively. The selection of appropriate boundary conditions depends on the nature of fluid–solid interactions and the physical properties of the channel walls. For macroscopic flows over smooth, hydrophilic surfaces, the classical no-slip condition is generally valid, as the fluid adheres completely to the solid boundary. This condition represents a limiting case of the more general Navier slip formulation, in which the slip length is zero.

When partial slip occurs due to surface characteristics such as hydrophobic coatings, microscale roughness, or rarefaction effects, Navier slip boundary conditions become more appropriate. Symmetric Navier slip arises when both channel walls have identical slip lengths, typically corresponding to geometrically and materially similar surfaces. Conversely, asymmetric Navier slip occurs when the two walls exhibit different surface properties, such as one being hydrophilic and the other hydrophobic, or when different coatings or material treatments are applied.

The general Navier slip formulation adopted in this study encompasses all these cases, offering a unified and continuous framework for analysing the influence of wall conditions on flow stability. Such considerations are crucial in microfluidic systems, coated channels, and engineered surfaces, where wall slip can significantly affect overall flow behaviour.

### General navier-slip flow

The first set of boundary conditions corresponds to slip at both channel walls.

The boundary conditions for equations (7-9) are established as follows.

20a $$\begin{array}{c}u-{k}_{1}\frac{du}{dy}=0,v=0\,on\,y=-1\end{array}$$

20b $$\begin{array}{c}u+{k}_{2}\frac{du}{dy}=0,v=0\,on\,y=1\end{array}$$

Where ${k}_{1}$ and ${k}_{2}$ are the slip lengths at the lower and upper walls, respectively.

Hence, the steady, unidirectional base flow $\overline{u }\left(y\right)$ is given by

21 $$\begin{array}{c}\overline{u }\left(y\right)=\frac{A}{{M}^{2}}\left[1-\frac{\left({b}_{2}-{b}_{1}\right)}{{a}_{1}{b}_{2}-{a}_{2}{b}_{1}}\mathrm{cosh}\left(My\right)-\frac{{a}_{1}{b}_{2}-{a}_{2}{b}_{1}-{a}_{1}\left({b}_{2}-{b}_{1}\right)}{{b}_{1}\left({a}_{1}{b}_{2}-{a}_{2}{b}_{1}\right)}\mathrm{sinh}\left(My\right)\right]\end{array}$$

Where

$$c=\mathrm{cosh}\left(M\right),\hspace{1em}s=\mathrm{sinh}\left(M\right), {a}_{1}=c+{k}_{1}Ms,\hspace{1em}{b}_{1}=-s-{k}_{1}Mc$$

$${a}_{2}=c+{k}_{2}Ms,\hspace{1em}{b}_{2}=s+{k}_{2}Mc$$

The eigenvalue problem given by equation (18) is then solved subject to an equivalent set of boundary conditions.

22a $$\begin{array}{c}\phi =0,\hspace{1em}D\phi -{k}_{1}{D}^{2}\phi =0\hspace{1em}aty=-1\end{array}$$

22b $$\begin{array}{c}\phi =0, D\phi +{k}_{2}{D}^{2}\phi =0\hspace{1em}aty=1\end{array}$$

### No-slip flow

For the classical no-slip case, the parameters are set to ${k}_{1}={k}_{2}=0$ in equation (21),

23 $$\begin{array}{c}u=0, v=0\,on\,y=\pm 1\end{array}$$

The base velocity profile reduces to the classical MHD Poiseuille flow:

24 $$\begin{array}{c}\overline{u }\left(y\right)=\frac{A}{{M}^{2}}\left(1-\frac{\mathrm{cosh}\left(My\right)}{\mathrm{cosh}\left(M\right)}\right)\end{array}$$

As $M \to 0,$ we expand the hyperbolic functions using the Taylor series:

$$cosh\left(My\right)\approx 1+1/2{\left(My\right)}^{2}$$

$$cosh\left(M\right)\approx 1+1/2{M}^{2}$$

Substituting the series expansions gives

$$\overline{u }\left(y\right)=\frac{A}{{M}^{2}}\left(1-\frac{\left(1+\raisebox{1ex}{$1$}\!\left/ \!\raisebox{-1ex}{$2$}\right.{M}^{2}{y}^{2}\right)}{\left(1+\raisebox{1ex}{$1$}\!\left/ \!\raisebox{-1ex}{$2$}\right.{M}^{2}\right)}\right)$$

Expanding the denominator for small M

$$\frac{\left(1+\raisebox{1ex}{$1$}\!\left/ \!\raisebox{-1ex}{$2$}\right.{M}^{2}{y}^{2}\right)}{\left(1+\raisebox{1ex}{$1$}\!\left/ \!\raisebox{-1ex}{$2$}\right.{M}^{2}\right)}\approx 1+\raisebox{1ex}{$1$}\!\left/ \!\raisebox{-1ex}{$2$}\right.{\mathrm{M}}^{2}\left({\mathrm{y}}^{2}-1\right)$$

Thus,

$$\overline{u }\left(y\right)=\frac{A}{{M}^{2}}\left(1-( 1 + \raisebox{1ex}{$1$}\!\left/ \!\raisebox{-1ex}{$2$}\right.{\mathrm{M}}^{2} ({\mathrm{y}}^{2} - 1)\right)$$

25 $$\begin{array}{c}\overline{u }\left(y\right)=\frac{A}{2}\left(1-{y}^{2}\right)\end{array}$$

Equation (25) is the classical plane Poiseuille flow with no magnetic field.

The eigenvalue problem given by equation (18) is then solved subject to an equivalent set of boundary conditions.

26 $$\begin{array}{c}\phi ={0}^{\mathrm{o}}, D\phi ={0}^{\mathrm{o}} aty=\pm 1\end{array}$$

### Symmetric navier-slip flow

For symmetric Navier slip, we set ${k}_{1}={k}_{2} =k$ in equation (21) and apply the corresponding boundary conditions to equations (7-9).

27a $$\begin{array}{c}u-k\frac{du}{dy}=0,v=0\,on\,y=-1\end{array}$$

27b $$\begin{array}{c}u+k\frac{du}{dy}=0,v=0\,on\,y=1\end{array}$$

The corresponding steady base-flow velocity reduces to:

28 $$\begin{array}{c}\overline{u }\left(y\right)=\frac{A}{{M}^{2}}\left(1-\frac{\mathrm{cosh}\left(My\right)}{\mathrm{cosh}\left(M\right)+\mathrm{kMsinh}\left(\mathrm{M}\right)}\right)\end{array}$$

The eigenvalue problem given by equation (18) is then solved subject to an equivalent set of boundary conditions.

29a $$\begin{array}{c}\phi =0,\hspace{1em}D\phi -k{D}^{2}\phi =0\hspace{1em}aty=-1\end{array}$$

29b $$\begin{array}{c}\phi =0,D\phi +k{D}^{2}\phi =0\hspace{1em}aty=1\end{array}$$

### Asymmetric navier-slip flow

In this case, a slip is applied only at the lower wall. Accordingly, for asymmetric Navier slip, we set ${k}_{1}=k$ and ${k}_{2}=0$ in equation (21) and impose the corresponding boundary conditions for equations (7-9).

30a $$\begin{array}{c}u-k\frac{du}{dy}=0,v=0\,on\,y=-1\end{array}$$

30b $$\begin{array}{c}u=0,v=0\,on\,y=1\end{array}$$

The corresponding steady base-flow velocity is given by:

31 $$\begin{aligned} \overline{u }\left(y\right) &=\frac{A}{{M}^{2}}\left[1+\frac{2s+kMc}{2cs+kM\left({s}^{2}+{c}^{2}\right)}\mathrm{cosh}\left(My\right)\right. \\& \left. -\frac{\left(-s-kMc\right)\left[2cs+kM\left({s}^{2}+{c}^{2}\right)\right]}{2cs+kM\left({s}^{2}+{c}^{2}\right)-\left(c+kMs\right)\left(2s+kMc\right)}\mathrm{sinh}\left(My\right)\right]\end{aligned}$$

The eigenvalue problem given by equation (18) is then solved subject to an equivalent set of boundary conditions.

32a $$\begin{array}{c}\phi =0,\hspace{1em}D\phi -k{D}^{2}\phi =0\hspace{1em}at y=-1\#\end{array}$$

32b $$\begin{array}{c}\phi =0,\hspace{1em}D\phi =0\hspace{1em}at y=1\end{array}$$

## Results and discussion

A Chebyshev spectral collocation method is utilized to calculate the base flow velocity profiles $\overline{u }\left(y\right)$ for four different types of boundary conditions: no-slip, symmetric slip, asymmetric slip, and general Navier-slip. A pressure gradient influences the flow within a porous medium, as does a magnetic field. The presence of wall slips significantly changes the shape and magnitude of the base velocity profile. Slip conditions increase the velocity near the boundary and shift the location of the maximum velocity. Since asymmetric and slip affect the flow profile, symmetric slip maintains a symmetrical flow while increasing the flow rate. The decreasing effects of the magnetic parameter and the porous resistance parameter lower the entire velocity. These results emphasise the importance of boundary conditions in flow dynamics, particularly in applications involving microfluidic systems, porous media, and magnetohydrodynamic systems.

The Chebyshev spectral collocation method is also used to solve the governing equations and the related eigenvalue problem. In this approach, the governing equations are enforced at selected collocation points, and the solution variables are expanded in terms of Chebyshev polynomials. One of the main advantages of this method is its spectral accuracy, which enables highly accurate solutions with relatively few grid points. Moreover, its ability to efficiently handle higher-order derivatives and complex boundary conditions makes it particularly suitable for linear stability analysis, yielding accurate eigenvalues for stability predictions. Compared with finite difference and finite element methods, the Chebyshev spectral collocation method exhibits faster convergence and reduced numerical error, making it an effective and efficient tool for hydrodynamic and MHD flow problems.

In this study, the physical and dimensionless parameters are selected based on previously published research on wall slip effects and magnetohydrodynamic flow in porous channels. The chosen parameter ranges ensure both physical relevance and mathematical consistency, enabling a comprehensive investigation of the influence of wall slip, porous resistance, and magnetic field strength on flow stability.

Specifically, the Reynolds number is varied between 5000 and 10000 to capture inertial effects, while the Hartmann number ranges from 0 to 10 to represent different magnetic field intensities. The Darcy parameter, which characterises the porous medium’s resistance, is also varied between 0 and 10. To model no-slip, symmetric slip, and asymmetric slip conditions, the Navier slip lengths at the lower and upper walls are set to 0–1.0.0. The disturbance wave number is varied from 0.1 to 2.0, and the phase speed is obtained as a complex eigenvalue of the stability problem. The characteristic length scale is taken as the channel half-width, which is normalised to unity. These parameter values are consistent with earlier stability analyses of porous and magnetohydrodynamic channel flows, enabling a meaningful comparison with existing results.

### General navier-slip flow

In this most common configuration, differing slip lengths at the upper and lower walls result in a significantly modified velocity profile. The velocity increases toward the lower slip wall, as the upper wall with a larger slip length permits greater fluid motion. This setup represents situations involving graded material properties or differential surface treatments. Such conditions are critical in engineering applications that require precise control of flow paths, including porous reactors, MHD pumps, and systems with specially engineered channel walls.

The figures represent velocity profiles of fluid flow in a channel with varying slip lengths $\left({k}_{2}\right),$ Hartmann numbers $\left(H\right),$ and porosity parameters $\left(S\right),$ while maintaining a fixed lower wall slip length ${k}_{1}=0.01$. The Chebyshev spectral collocation method is employed with $N = 120$ collocation points, while the remaining parameters are fixed at $Re = 10000$, $\rm{\alpha }= 1.0,$ and $A = 2$. These plots illustrate how the combined effects of wall slip, magnetic field strength (Hartmann number), and porous medium resistance (porosity parameter $S$) influence the velocity distribution across the channel.

**Fig. 2:**
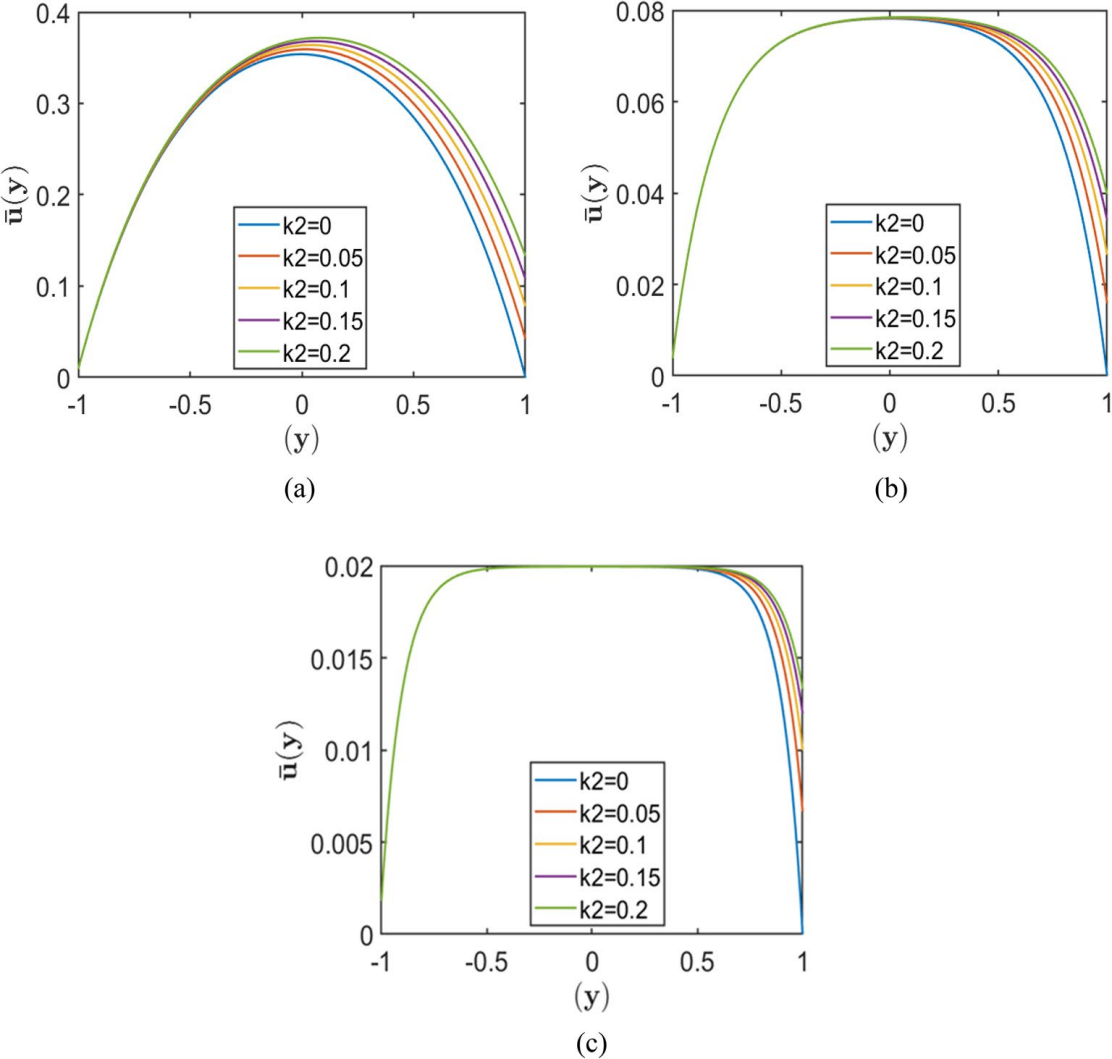
Velocity profiles for different slip lengths ${k}_{2}$, with fixed ${k}_{1}=0.01$, and porosity parameters $S = 0.5$ corresponding to Hartmann numbers (**a**) $H = 2.0$, (**b**) $H = 5.0,$ and (**c**) $H = 10.0$.

**Fig. 3:**
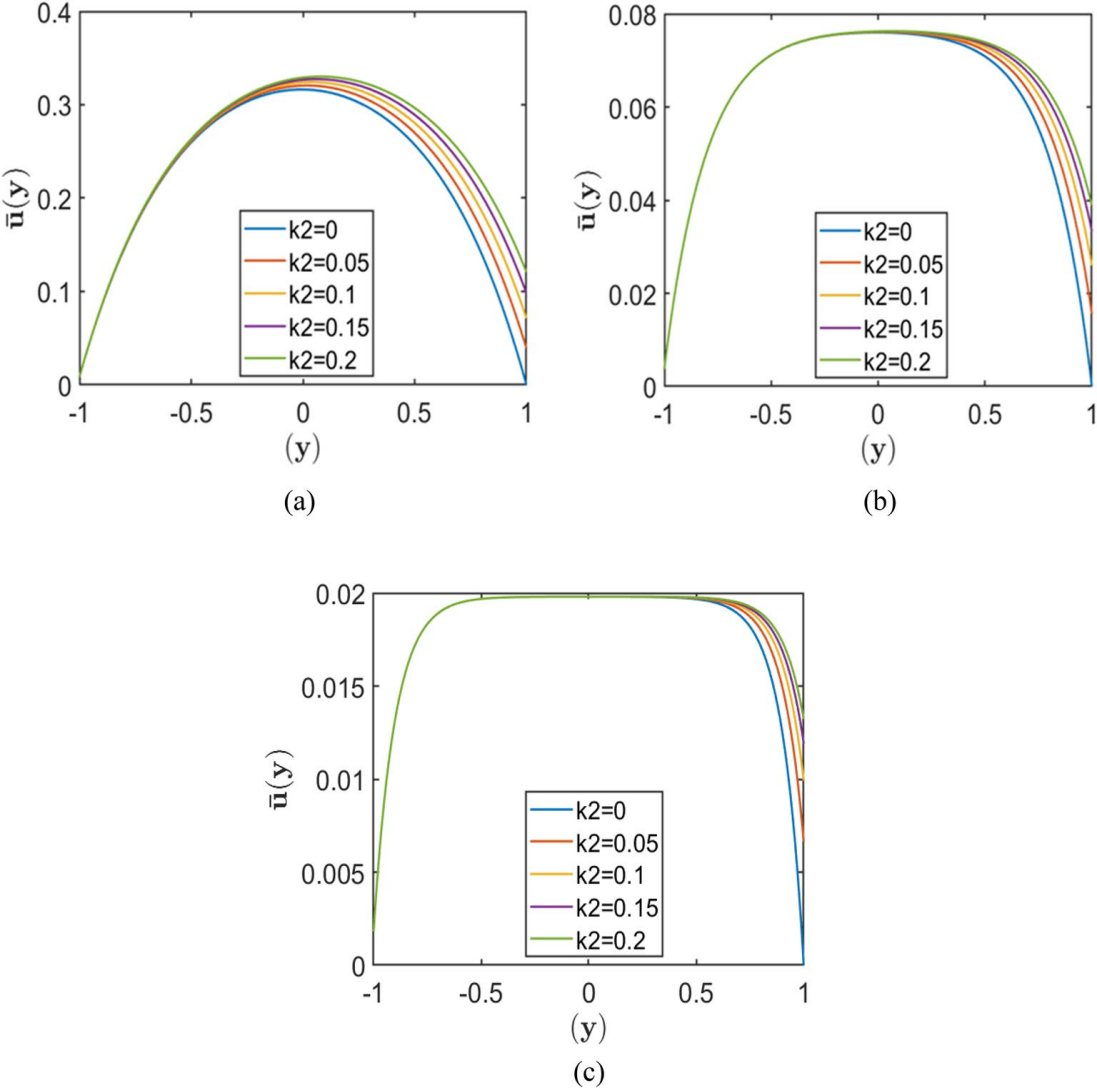
Velocity profiles for different slip lengths ${k}_{2}$, with fixed ${k}_{1}=0.01$, and porosity parameters $S = 1.0$ corresponding to Hartmann numbers (**a**) $H = 2.0$, (**b**) $H = 5.0,$ and (**c**) $H = 10.0$.

**Fig. 4:**
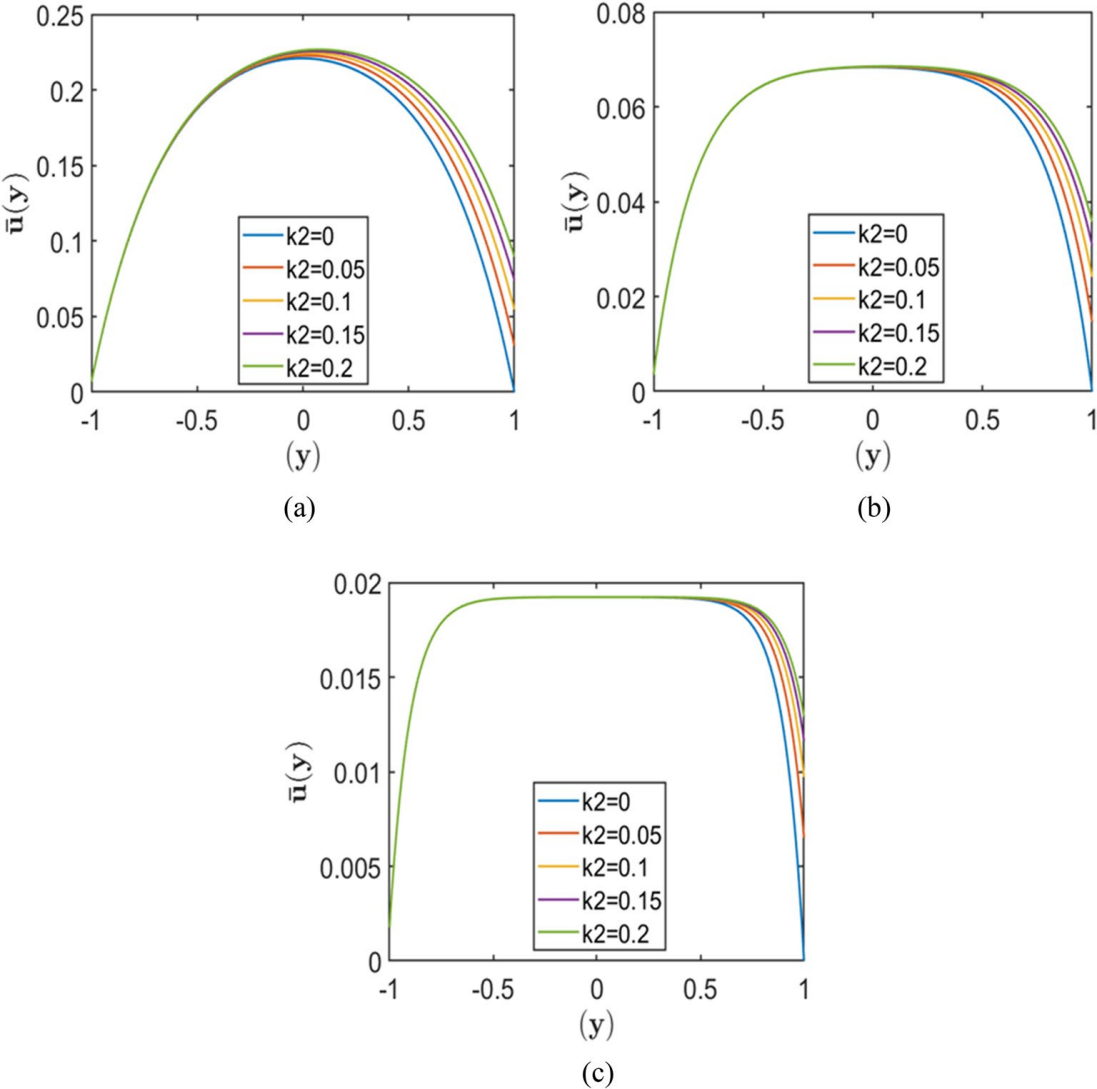
Velocity profiles for different slip lengths ${k}_{2}$, with fixed ${k}_{1}=0.01$, and porosity parameters $S = 2.0$ corresponding to Hartmann numbers (**a**) $H = 2.0$, (**b**) $H = 5.0,$ and (**c**) $H = 10.0$.

Figures 2, 3, and 4 each comprise three subplots (a, b, c) corresponding to increasing Hartmann numbers $\left(H=2, 4, 10\right)$, obtained by varying the upper wall slip length ${k}_{2}$ while keeping ${k}_{1}$ constant. Each figure represents a different value of the porosity parameter, $=0.5, 1.0,$ $2.0$, respectively. In all plots, the horizontal axis denotes the normalised channel height (wall-normal direction $y$), while the vertical axis represents the dimensionless velocity.

The results indicate that increasing the upper wall slip length ${k}_{2}$ consistently enhances the near-wall velocity, with the most pronounced effect occurring at lower Hartmann numbers and smaller porosity parameters. As the Hartmann number increases, the velocity profiles become progressively flatter, and the peak velocity decreases, demonstrating the strong damping effect of the magnetic field on fluid motion. Similarly, higher values of the porosity parameter $S$ reduce the overall velocity due to increased resistive drag from the porous medium. As a result, the influence of wall slip weakens at larger $S$, where porous resistance becomes the dominant factor governing the flow behaviour.

### No-slip flow

Under the classical no-slip condition, the velocity at both channel walls is strictly zero, resulting in a symmetric parabolic profile with the mid-plane $\left(y=0\right)$ as the centreline. Compared with the standard MHD-Poiseuille flow, the combined effects of the magnetic field and porous drag substantially reduce the maximum velocity. This case corresponds to the lowest volumetric flow rate and the highest wall shear among the configurations considered, and therefore serves as a reference baseline for comparison.

**Fig. 5:**
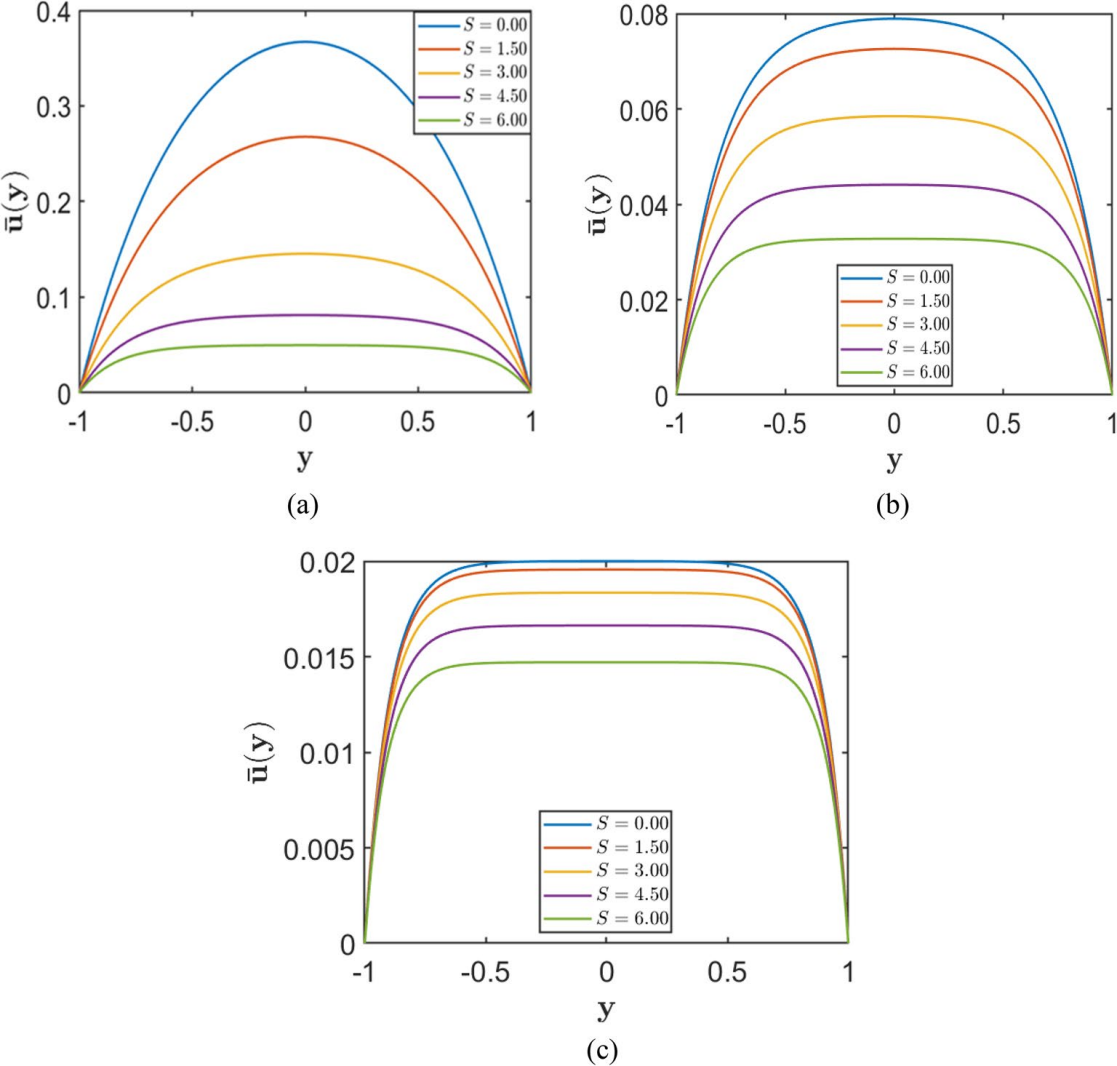
Velocity profiles for varying porosity parameter $S=(0.0, 1.5, 3.0, 4.5, 6.0 )$, corresponding to fixed Hartmann numbers (**a**) $H = 2.0$, (**b**) $H = 5.0$, and (**c**) $H = 10.0$.

**Fig. 6:**
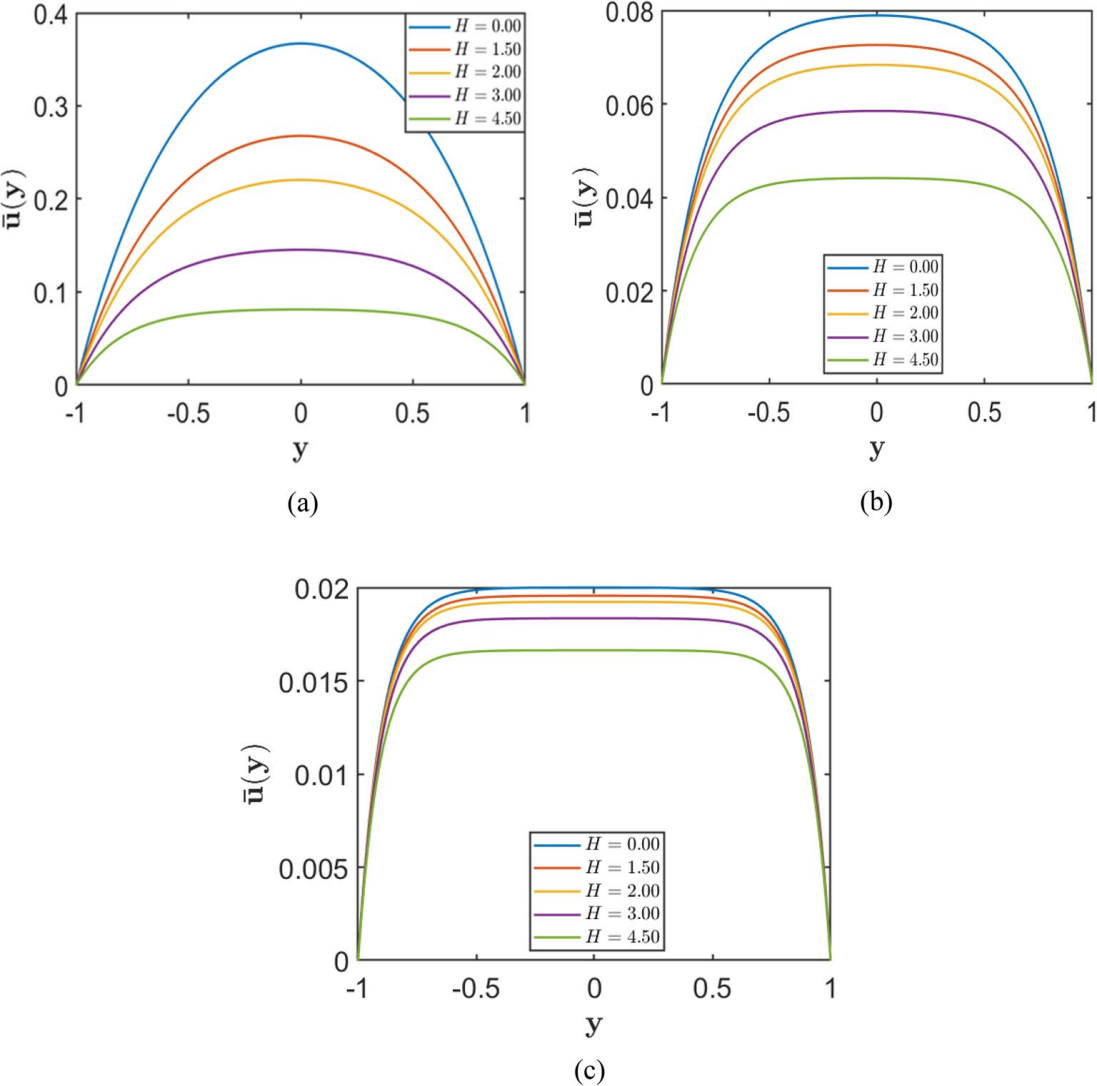
Velocity profiles for varying Hartmann number $H=(0.0, 1.5, 2.0, 3.0, 4.5)$, corresponding to fixed porosity parameters (**a**) $S = 2.0$, (**b**) $S = 5.0$, and (**c**) $S = 10.0$.

Figures 5 and 6 illustrate the influence of the porosity parameter $S$ and the Hartmann number $H$ on the velocity profiles in a channel subject to no-slip boundary conditions. The Chebyshev spectral collocation method is applied using $N=120$ collocation points, while the remaining parameters are fixed at $Re=10000$, $\alpha =1.0$, and $A=2$.

Figure 5 presents velocity profiles for different values of $S$ at fixed Hartmann numbers $\left(H=2, 5, 10\right)$. In all three subplots, increasing $S$ reduces the maximum velocity at the channel centre and shifts the entire velocity profile downward, reflecting the increased resistance introduced by the porous medium. Furthermore, as $H$ increases from subplot (a) to (c), this damping effect becomes more pronounced, indicating that stronger magnetic fields further suppress fluid motion throughout the channel.

Conversely, Figure 6 depicts the effect of varying the Hartmann number $H$ at fixed values of the porosity parameter $\left(S=2, 5, 10\right)$. In each subplot, an increase in $H$ results in a consistent decrease in the central velocity and a progressively flatter velocity profile, particularly at higher values of $H$. The stronger magnetic influence, acting in conjunction with porous resistance, leads to a substantial reduction in fluid velocity across the channel.

Taken together, these results demonstrate that both porous drag (associated with higher $S$) and magnetic damping (associated with higher $H$) play significant roles in reducing the flow velocity. Their combined effects yield low and relatively uniform velocity distributions within the channel, especially when both parameters are substantial.

### Symmetric navier-slip flow

**Fig. 7:**
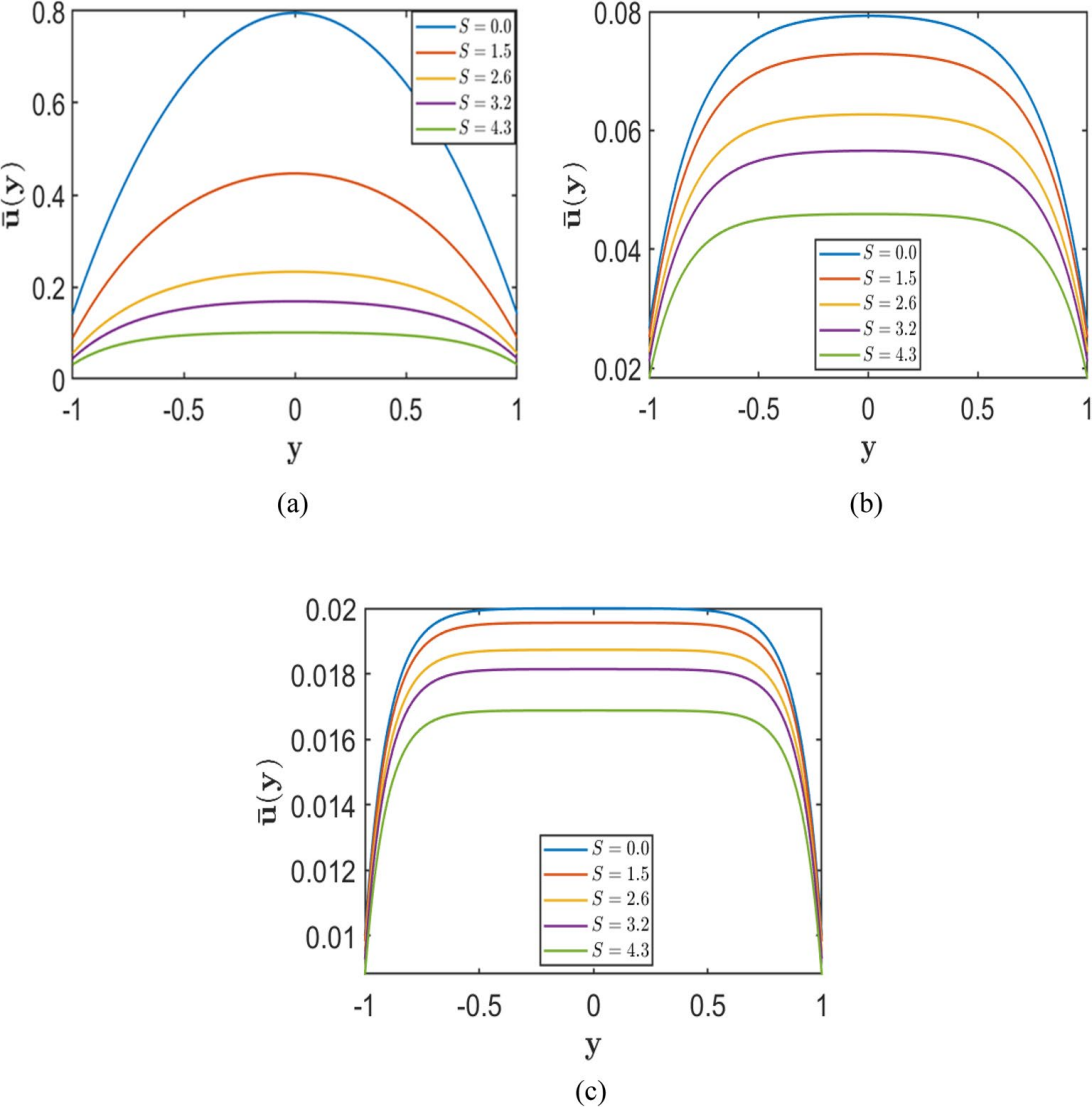
Velocity profiles for varying porosity parameter $S=(0.0, 1.5, 2.6, 3.2, 4.3)$, with fixed slip length k=0.1 corresponding to fixed Hartmann numbers (**a**) $H = 1.0$, (**b**) $H = 5.0$, and (**c**) $H = 10.0$.

**Fig. 8:**
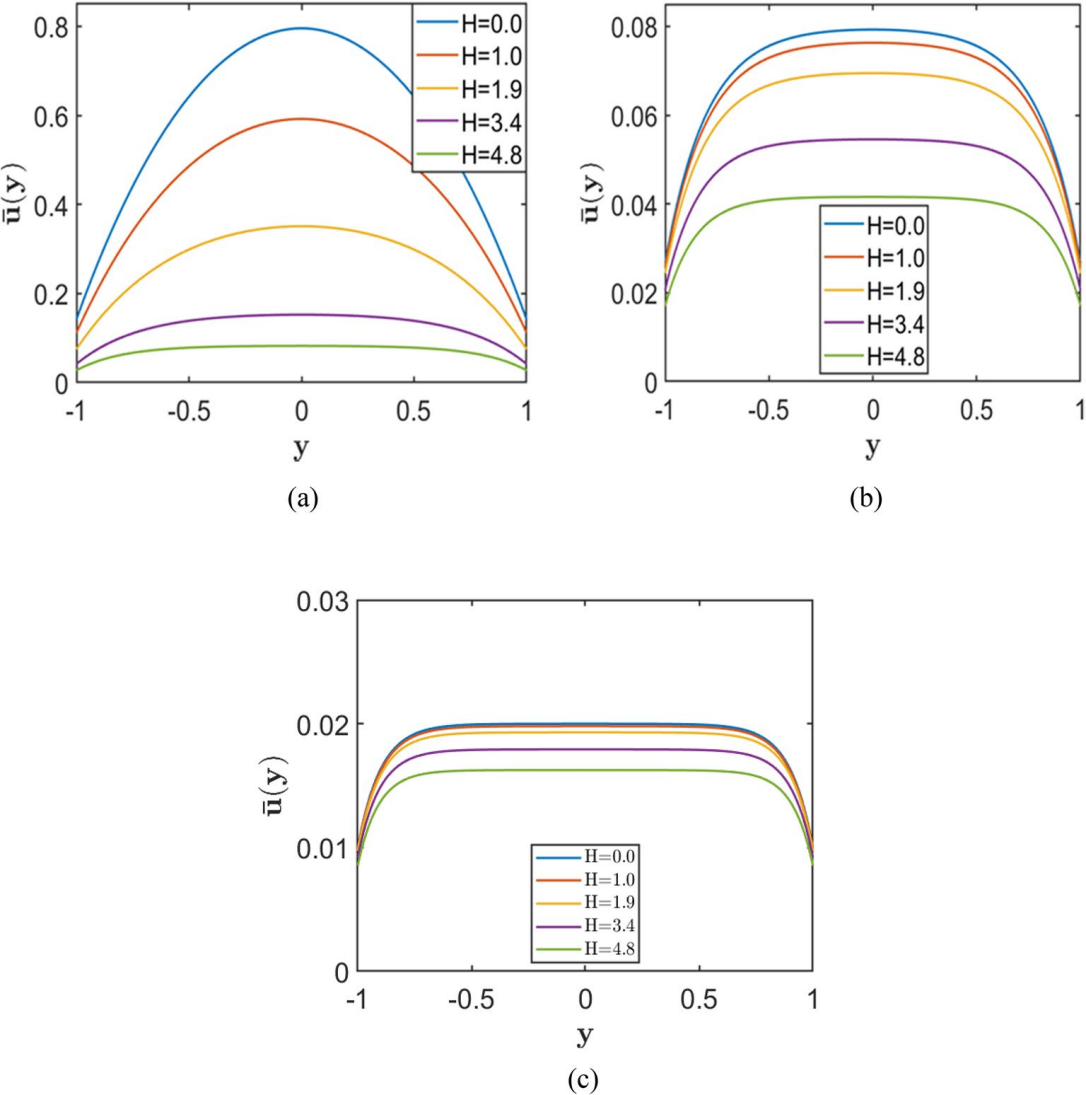
Velocity profiles for varying Hartmann number $H=(0.0, 1.0, 1.9, 3.4, 4.8)$, with fixed slip length k=0.1 corresponding to fixed Porosity parameter (**a**) $S = 1.0$, (**b**) $S = 5.0$, and (**c**) $S = 10.0$.

**Fig. 9:**
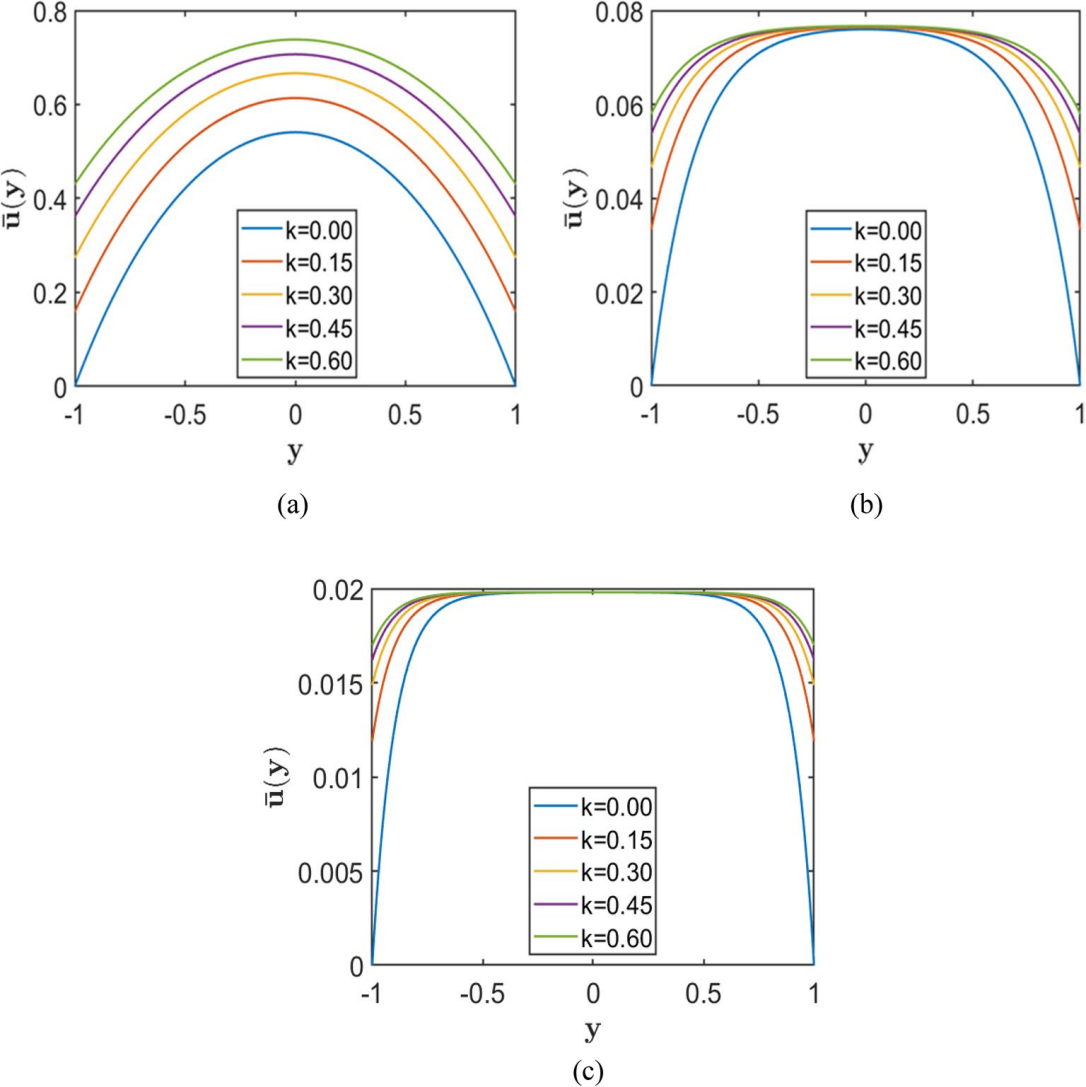
Velocity profiles for varying slip length $k=(0.0, 0.15, 0.3, 0.45, 0.6),$ with fixed Hartmann number $H$=1, corresponding to fixed Porosity parameter (**a**) $S = 1.0$, (**b**) $S = 5.0$, and (**c**) $S = 10.0$.

Equal slip at both walls produces a flatter yet symmetric velocity profile. The presence of non-zero velocities at the boundaries reduces wall shear stress, allowing a higher base velocity and a greater overall flow rate than in the no-slip condition. The Chebyshev spectral collocation method is employed with $N =120$ collocation points, while the remaining parameters are fixed at $Re = 10000$, $\rm{\alpha }= 1.0,$ and $A = 2$. This type of flow is beneficial in applications that require uniform flow improvement, such as lubrication systems and biomedical devices, where symmetric boundary conditions minimise energy loss while maintaining an equal distribution. Figures 7, 8, and 9 present the effects of the porosity parameter $\left(S\right),$ Hartmann number $\left(H\right),$ and symmetric Navier-Slip length $\left(k\right)$ on the velocity profiles in the channel.

Figures 7, 8, and 9 show that, at fixed values of $H$ and $k$, increasing the porosity parameter $S$ results in a marked reduction in the maximum velocity and a progressive flattening of the velocity profile, underscoring the strong damping effect imposed by the porous medium. The corresponding results further indicate that, for fixed $S$ and $k$, an increase in the Hartmann number $H$ reduces both the central and wall velocities, yielding more uniform velocity distributions across the channel. This behaviour reflects the growing influence of magnetic damping on the flow.

In addition, the results demonstrate that increasing the symmetric slip length $k$ at fixed $S$ and for different values of $H$ enhances the velocity both near the walls and at the channel centre. However, this slip-induced enhancement diminishes as the magnetic field strength increases, since the velocity profiles tend to converge and become progressively flatter at higher $H$.

Overall, these findings indicate that although symmetric wall slip can substantially increase the flow velocity, its influence is weakened by strong porous resistance and magnetic effects. The highest velocities are therefore achieved under conditions of low porosity parameter $S$, low Hartmann number $H$, and sufficiently large slip length $k$.

### Asymmetric navier-slip flow

In this configuration, slip is imposed only at the lower wall, which breaks the symmetry of the velocity profile. As a result, the peak velocity shifts upward toward the no-slip wall, indicating enhanced flow near the slipped boundary. Such asymmetric flow behaviour is beneficial for applications that require directional control, such as particle sorting and directed transport in microchannels. It also represents practical situations in which channel walls possess different surface properties or coatings.

**Fig. 10:**
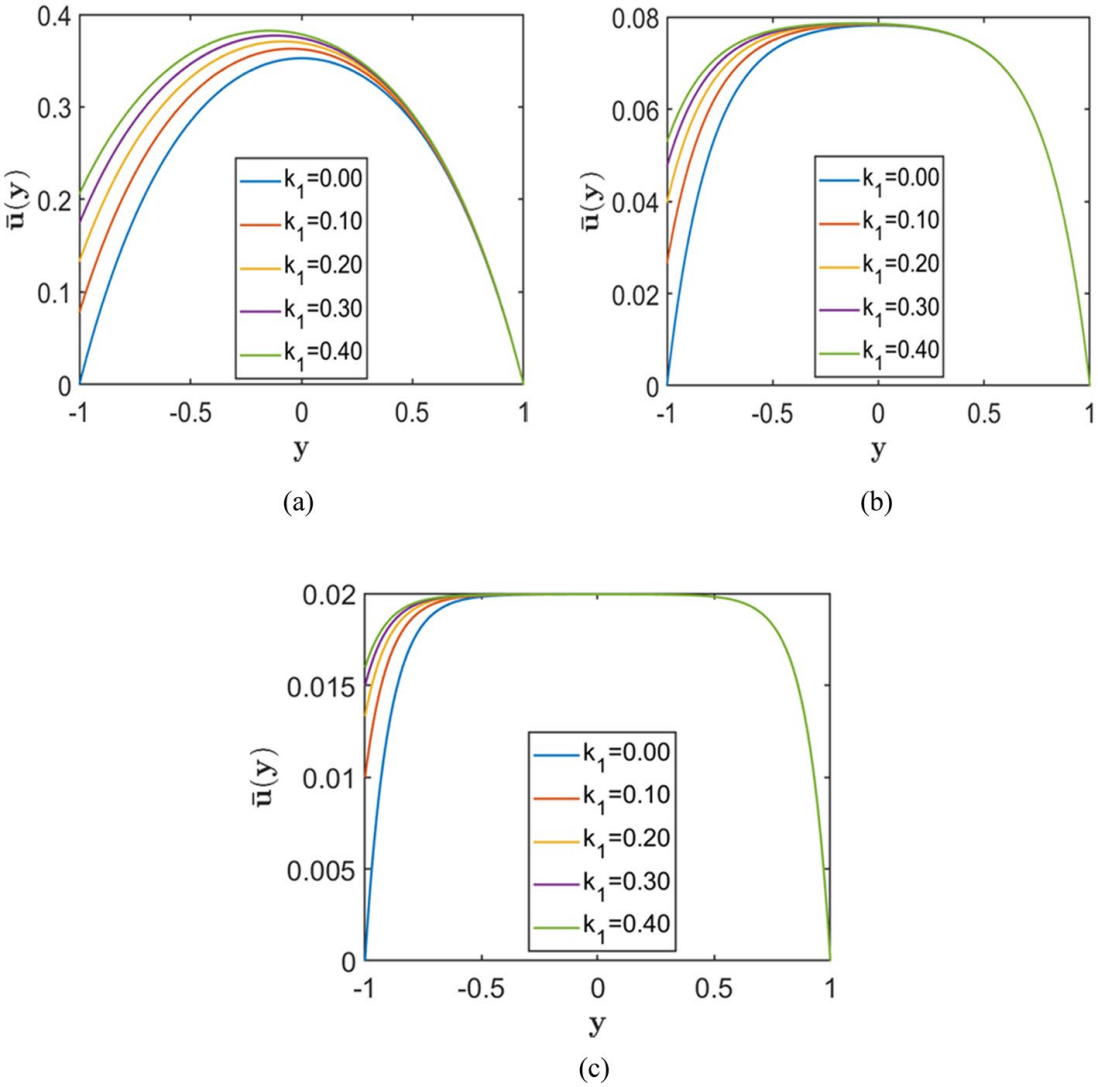
Velocity profiles for different slip lengths ${k}_{1}=(0.0, 0.1, 0.2, 0.3, 0.4)$, with porosity parameter $S=0.5$ corresponding to fixed Hartmann numbers (**a**) $H = 2.0$, (**b**) $H = 5.0$, and (**c**) $H = 10.0$.

**Fig. 11:**
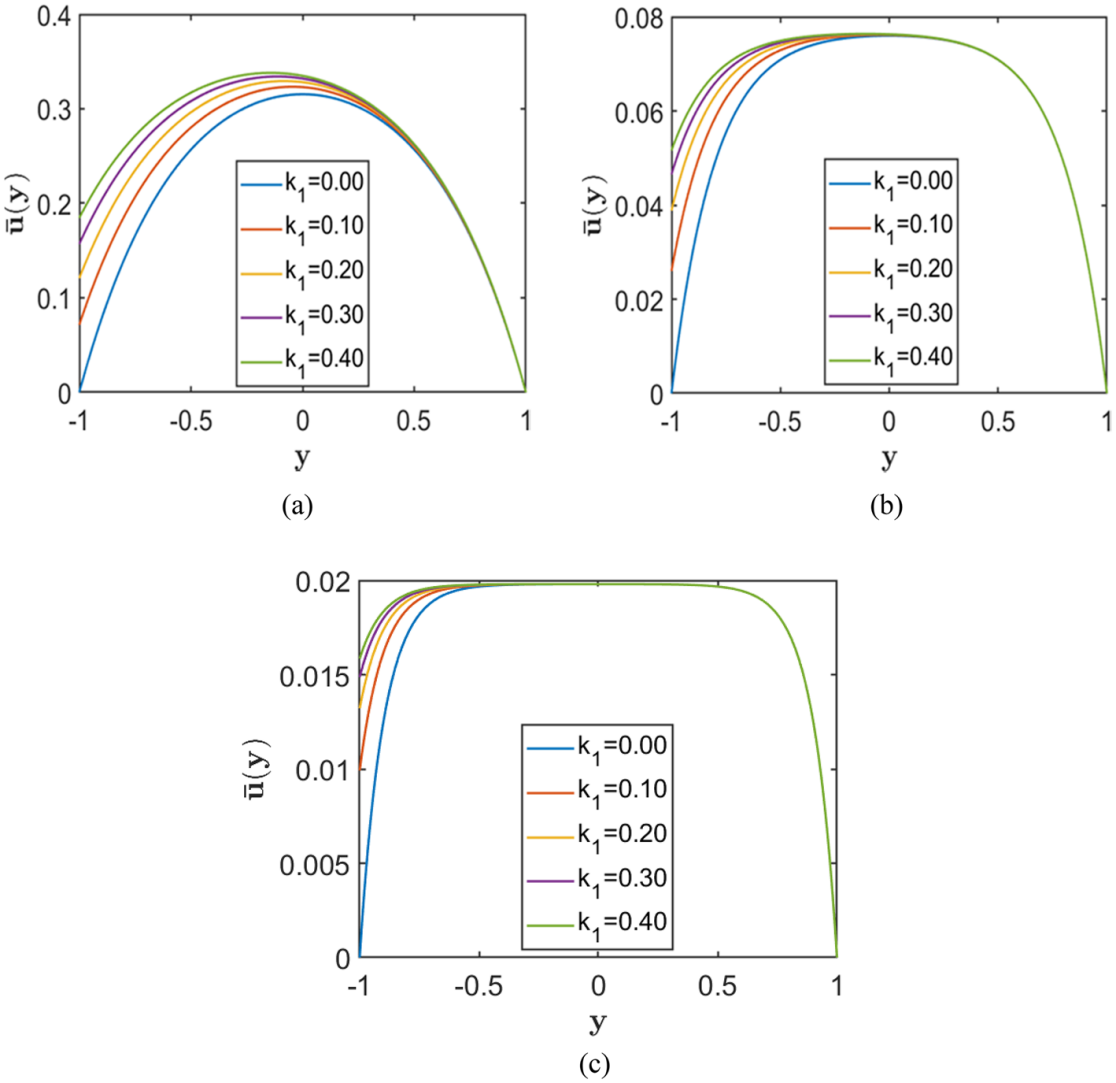
Velocity profiles for different slip lengths ${k}_{1}=(0.0, 0.1, 0.2, 0.3, 0.4)$, with porosity parameter $S=1.0$ corresponding to fixed Hartmann numbers (**a**) $H = 2.0$, (**b**) $H = 5.0$, and (**c**) $H = 10.0$.

**Fig. 12:**
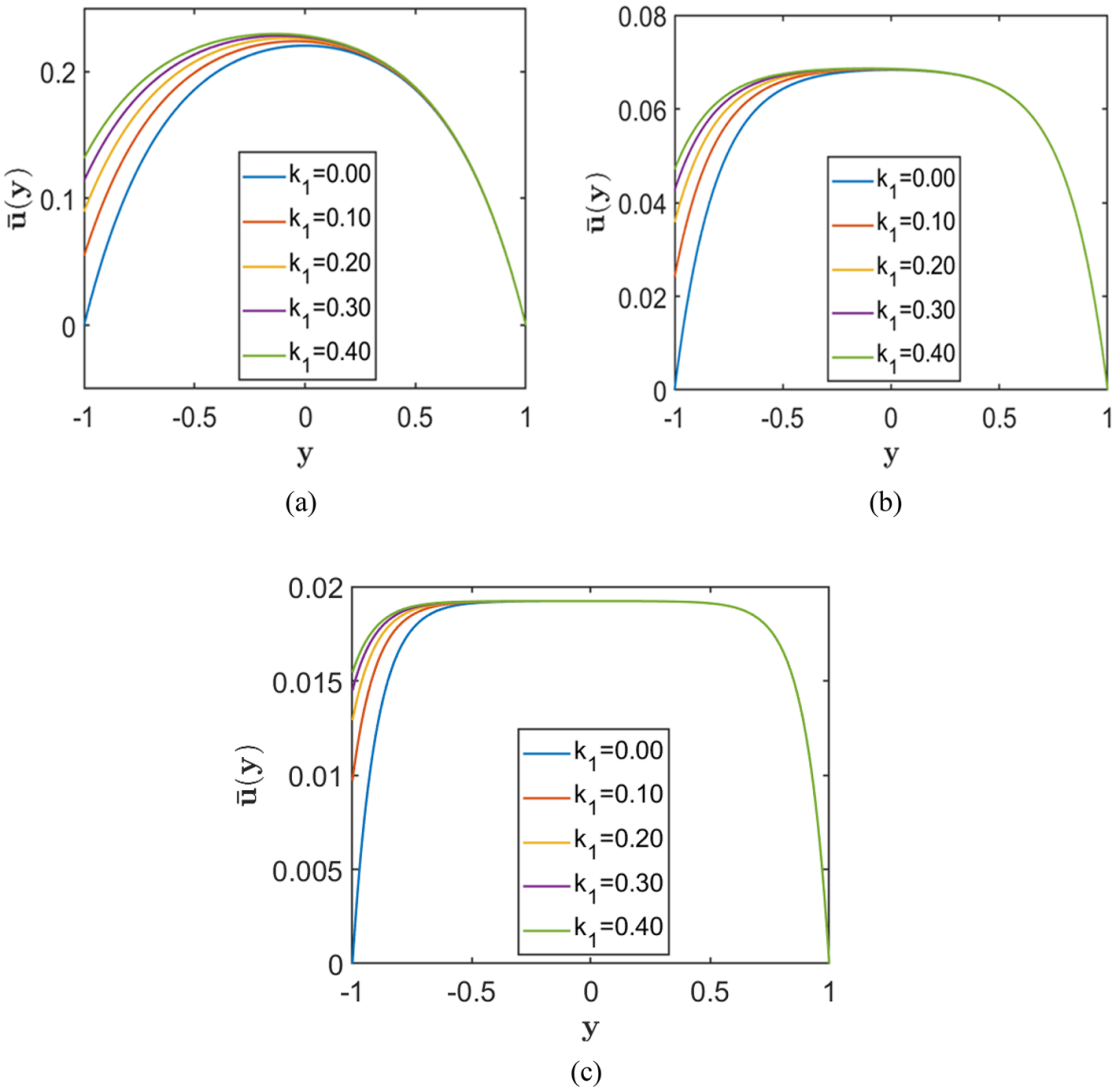
Velocity profiles for different slip lengths ${k}_{1}=(0.0, 0.1, 0.2, 0.3, 0.4)$, with porosity parameter $S=2.0$ corresponding to fixed Hartmann numbers (**a**) $H = 2.0$, (**b**) $H = 5.0$, and (**c**) $H = 10.0$.

Figures 10, 11, and 12 illustrate the effect of asymmetric Navier slip, where slip is applied exclusively at the lower wall, while the upper wall remains subject to the no-slip condition. Each figure corresponds to a fixed value of the porosity parameter $S=0.5, 1.0, 2.0$, respectively, arranged from top to bottom. Within each figure, the subplots illustrate the effect of increasing the Hartmann number $\left(H=2, 5, 10\right)$ on the velocity profiles. The Chebyshev spectral collocation method is used with $N=120$ collocation points, and the remaining parameters are held constant at $Re=10000$, $\alpha =1.0$, and $A=2$.

The results show that increasing the slip length at the lower wall enhances the velocity near that wall and elevates the overall velocity profile. However, this effect is more prominent at lower values of $H\,and\,S$. As the magnetic field strength or porosity parameter increases, the velocity profiles become flatter, and the influence of slip becomes negligible. Therefore, while asymmetric slip promotes higher flow rates under weak magnetic fields or low porous resistance, its impact diminishes considerably in channels with strong magnetic fields or highly resistive porous media.

### Comparative analysis of slip conditions

**Fig. 13:**
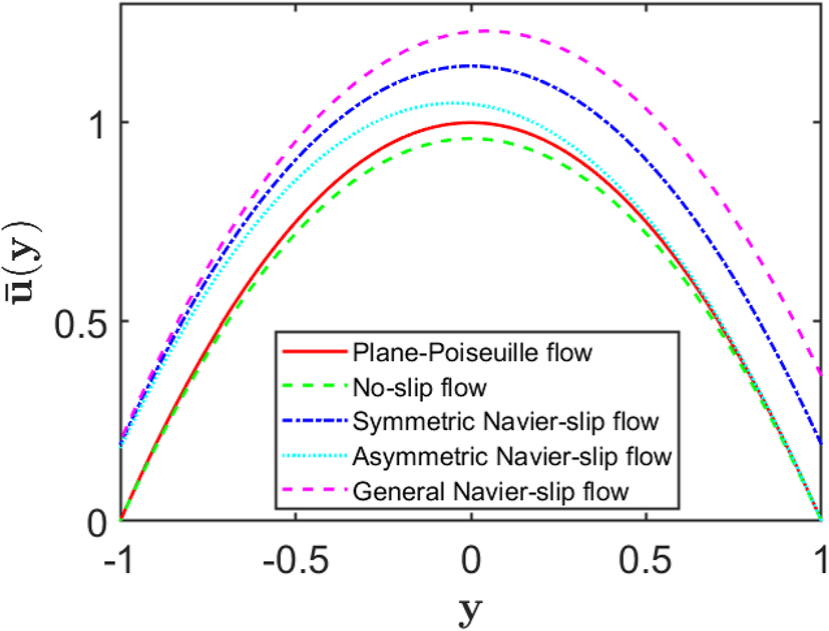
Velocity profiles across the channel for the present flow configuration at $Re=\mathrm{10,000}$, $\alpha =1.0$, $S=0.1$, $H=0.3$, $A=2$, and $N=120$.

Figure 13 shows the velocity profiles $\overline{u }(y)$ for different boundary conditions in the magnetohydrodynamic flow of a viscous incompressible fluid through a porous channel. The influence of Navier slip at the channel walls is clearly demonstrated by comparing the plane Poiseuille flow with the no-slip case and with symmetric, asymmetric, and general slip conditions. As expected, the classical no-slip flow yields the lowest velocity across the channel due to strong wall friction, while increasing slip lengths reduce this resistance and result in higher velocity magnitudes. The asymmetric slip case produces a slightly skewed velocity profile, indicating that unequal slip at the channel walls alters the flow symmetry. The general Navier-slip condition exhibits the highest peak velocity, highlighting the significant enhancement of flow rate when slip effects dominate. Overall, the figure emphasises the role of slip boundary conditions in modifying the flow structure in porous, magnetically influenced channels.

The slip lengths at the lower and upper walls are fixed as ${k}_{1}=0.1$ and ${k}_{2}=0.2$, respectively.

Table 1 presents the computed complex wave speed $c={c}_{r}+i{c}_{i}$ for $Re = 6000$, $\rm{\alpha }= 1.0$, and collocation points $N = 50 to 200$, under different wall boundary conditions. The real part of the wave speed, ${c}_{r}$ represents the phase velocity of disturbances, while the imaginary part, ${c}_{i}$ indicates the temporal growth rate (instability if ${c}_{i}>0$, damping if ${c}_{i}<0$). For the classical Poiseuille flow, the wave speed converges to $c \approx 0.259818 + 0.000323i$ as $N$ increases, which is consistent with benchmark results reported by Orszag^34^, thereby validating the numerical method. The positive ${c}_{i}$ indicates weak temporal instability, in agreement with the classical Orr-Sommerfeld analysis described by Drazin and Reid^32^.

**Table 1:** Computed complex wave speed$c={c}_{r}+i{c}_{i}$for$Re=6000, \alpha =1.0,\,and\,N=50\,to\,200$.

Flow	${k}_{1}$	${k}_{2}$	$k$	$S$	$H$	$N$	$c$
Poiseuille	0	0	0	0	0	50 100 150 200	0.2598158 + 0.0003231i 0.2598158 + 0.0003230i 0.2598158 + 0.0003230i 0.2598158 + 0.0003230i
No-slip	0	0	0	0.1	0.3	50 100 150 200	0.3360626 - 0.0025943i 0.3360626 - 0.0025944i 0.3360626 - 0.0025944i 0.3360626 - 0.0025944i
Symmetric navier-slip	k	k	0.1	0.1	0.3	50 100 150 200	0.3862944 + 0.0136482i 0.3862936 + 0.0136494i 0.3862936 + 0.0136494i 0.3862936 + 0.0136494i
Asymmetric navier-slip	k	0	0.1	0.1	0.3	50 100 150 200	0.2218792 - 0.0094705i 0.2218774 - 0.0094659i 0.2218774 - 0.0094659i 0.2218774 - 0.0094659i
General navier-slip	0.1	0.2	0	0.1	0.3	50 100 150 200	0.5469271 - 0.0090788i 0.5469295 - 0.0090776i 0.5469295 - 0.0090776i 0.5469295 - 0.0090776i

In the no-slip condition, $\left(S=0.1, H=0.3\right)$, the eigenvalue changes to around $c \approx 0.336066 - 0.002594i$ when porous medium resistance is included. The results show that porous drag efficiently reduces disturbance amplification, and the negative imaginary component of $c$ implies that the porous material stabilises the flow. The eigenvalue in the symmetric Navier-slip case, when both walls have equal slip $\left({k}_{1}={k}_{2}=0.1\right),$ is $c \approx 0.386294 + 0.013647i$, where ${c}_{i}>0$ indicates instability. Slip increases the buildup of disturbances by decreasing wall shear and viscous dissipation. In contrast, when a slip is applied asymmetrically only at the lower wall, the eigenvalue is $c \approx 0.221877 - 0.009467i$, where the negative ${c}_{i}$ indicates stabilisation despite the altered velocity distribution. Similarly, for the general Navier-slip case with unequal slip (${k}_{1}=0.1,{k}_{2}=0.2$), the wave speed increases significantly to $c \approx 0.546929 - 0.009078i,$ but the negative growth rate indicates stable behaviour. Overall, the results show that while symmetric slip promotes instability by reducing shear resistance, both porous drag and asymmetric or unequal slip act to stabilise the flow. The consistency of results across different values of $N$ confirms the spectral accuracy and convergence of the Chebyshev spectral collocation method.

Table 2 presents the critical stability parameters for different flow configurations, including plane Poiseuille flow, no-slip porous flow, symmetric Navier-slip, asymmetric Navier-slip, and general Navier-slip conditions, with ${k}_{1}=k=0.1,{ k}_{2}=0.2, S=0.1, H=0.3,\,and\,N=120.$ The table presents the critical Reynolds number ($R{e}_{c}$), the corresponding critical wavenumber $({\rm{\alpha }}_{c}$), and the critical complex wave speed $\left(c={c}_{r}+i{c}_{i}\right)$. At neutral stability, the growth rate vanishes $\left({c}_{i}=0\right)$, which is confirmed for all cases in the table. For the classical plane Poiseuille flow, the critical values $R{e}_{c}\approx 5772.2,{\rm{\alpha }}_{c}\approx 1.02,and{C}_{r}\approx 0.263997$ agrees well with benchmark results reported by Orszag^34^, thereby validating the numerical approach. When porous medium resistance is introduced (no-slip porous case), the critical Reynolds number increases to $R{e}_{c}\approx 6799.1$, indicating that the porous medium stabilises the flow by requiring a higher Reynolds number for instability. In contrast, symmetric Navier-slip reduces the critical Reynolds number to $R{e}_{c}\approx 5875.4$, showing that the slip at both walls destabilises since reduced wall friction promotes disturbance growth. For the asymmetric Navier-slip case, the critical Reynolds number increases significantly to $R{e}_{c}\approx 7100.0$, highlighting the stabilising effect of slip applied at only one boundary. The general Navier-slip case with unequal slip lengths $\left({k}_{1}=0.1,{k}_{2}=0.2\right)$ yields $R{e}_{c}\approx 6187.9$, representing intermediate behaviour between the symmetric and asymmetric slip limits.

**Table 2:** Critical values for different flow configurations with ${k}_{1}=k=0.1,\,{k}_{2}=0.2,\,S=0.1,\,H=0.3,\,N=120.$

Flow type	$R{e}_{c}$	${\rm{\alpha }}_{c}$	${c}_{c}$
Plane-poiseuille	5772.2283	1.02052	0.263997 + 0.000000i
No-slip	6799.1094	1.0789	0.3387695 + 0.000000i
Symmetricn avier-slip	5875.40	1.18	0.3963423 + 0.000000i
Asymmetric navier-slip	7100.0381	0.80	0.2159968 + 0.000000i
General navier-slip	6187.850	0.68	0.4313619 + 0.000000i

Table 2 indicates that porous drag and asymmetric slip tend to stabilise the flow, whereas symmetric slip strongly destabilises it. The effect of general slip lies between these two extremes, depending on the relative slip lengths at the channel walls.

To validate the present analysis, the obtained stability results are compared with previously published studies on classical plane Poiseuille flow and slip-dependent channel flows. The critical Reynolds number for plane Poiseuille flow obtained in this study shows excellent agreement with Orszag’s^34^ benchmark results. Furthermore, the observed stabilizing influence of the magnetic field and porous resistance is consistent with earlier investigations of magnetohydrodynamic channel flows. The destabilizing effect of symmetric slip and the stabilizing behaviour of asymmetric slip are consistent with previously reported slip-flow stability analyses. These comparisons confirm the accuracy and reliability of the present numerical formulation.

In this study, the Reynolds number is treated as the primary bifurcation parameter governing the onset of instability, while the Hartmann and Darcy numbers serve as control parameters that shift the critical Reynolds number by modifying the effects of magnetic damping and porous resistance.

### Temporal stability characteristics

The temporal stability of a porous medium subjected to a transverse magnetic field during parallel channel flow is analysed by solving the generalized Orr-Sommerfeld eigenvalue problem. The eigenvalues are represented in terms of the complex phase speed, as indicated in equation (19).

For the representative case of Reynolds number $Re = 10000$ and streamwise wavenumber $\rm{\alpha }= 1.0$, with porosity parameter $S = 0.1$, magnetic parameter $H = 0.3$, and the Darcy resistance parameter $A = 2$, the eigenspectrum is computed using the Chebyshev spectral collocation method with $N = 120$ collocation points.

The computed spectra exhibit the typical organization into wall and centre branches, with eigenvalues aligned along characteristic curves in the complex plane. All eigenvalues fall in the lower half-plane $\left({c}_{i}<0\right)$, indicating temporally damped modes for this parameter set. The porosity parameter $S$ modifies the effective permeability of the medium, introducing additional damping compared to a clear fluid channel. The magnetic parameter $H$ contributes to Lorentz-force damping, while Darcy resistance $A$ shifts the branches further downward, thereby enhancing the system’s overall stability.

**Fig. 14:**
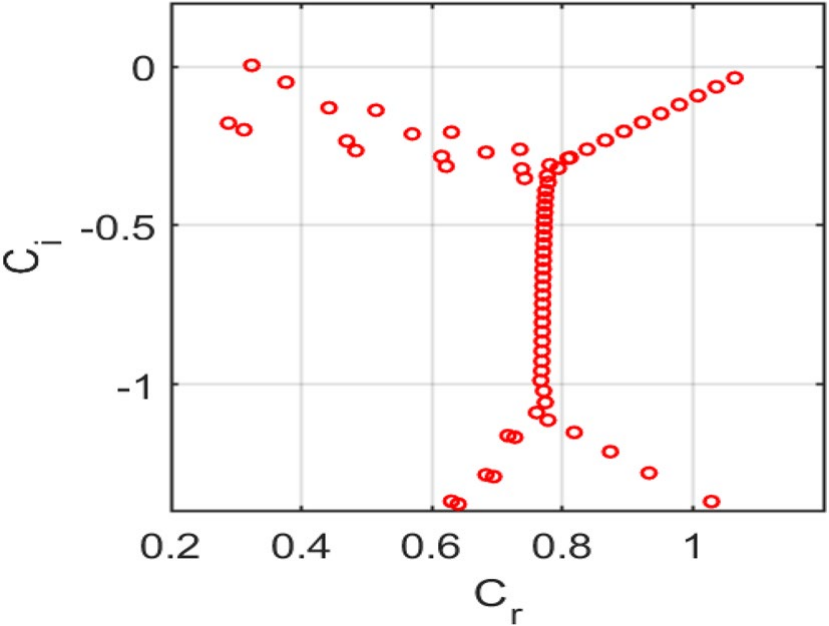
Eigenvalue spectrum for the plane-Poiseuille flow with $S=0\,and\,H=0$.

Figure 14 shows the** e**igenvalue spectrum of plane-Poiseuille flow with $S=0\,and\,H=0$, showing unstable modes $\left({c}_{i}>0\right),$ consistent with classical Tollmien-Schlichting instability*.*

**Fig. 15:**
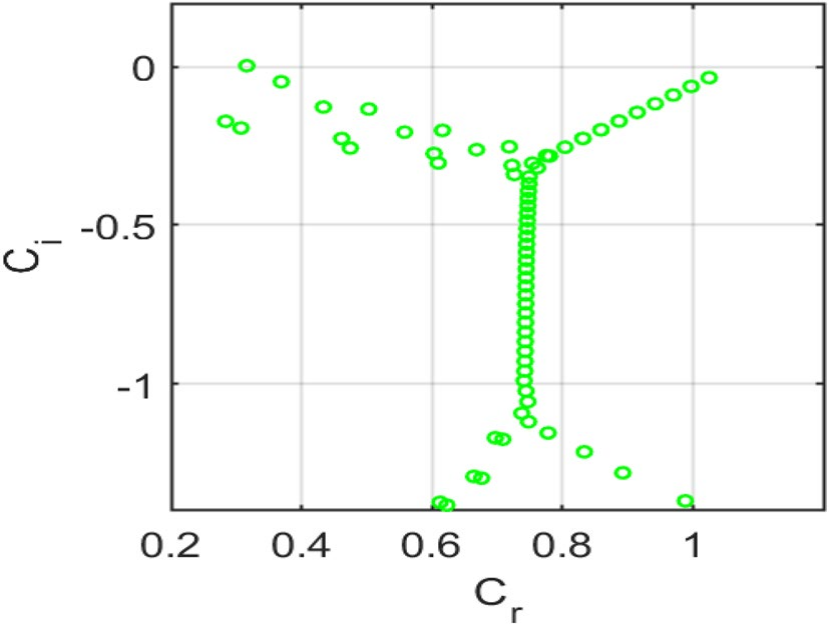
Eigenvalue spectrum for the no-slip flow ${k}_{1}={k}_{2}=0$.

**Fig. 16:**
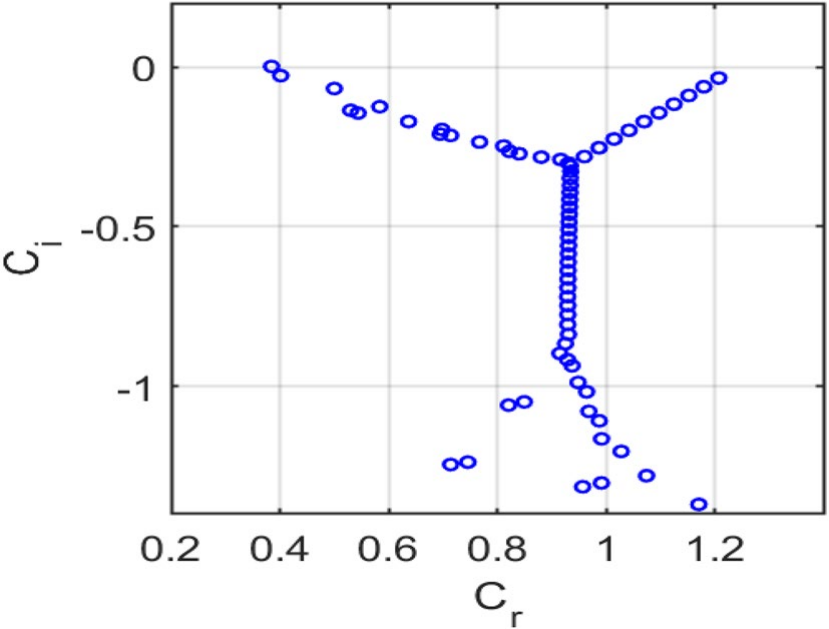
Eigenvalue spectrum for the symmetric Navier-slip flow with slip length ${k}_{1}={k}_{2}=0.1$.

**Fig. 17:**
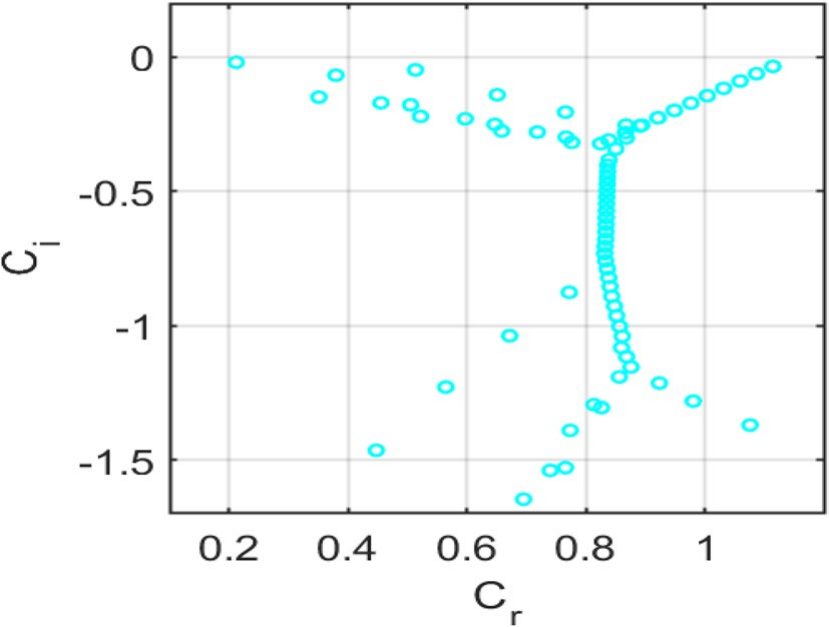
Eigenvalue spectrum of the asymmetric Navier-slip flow with slip length ${k}_{1}=0.1$, ${k}_{2}=0$.

**Fig. 18:**
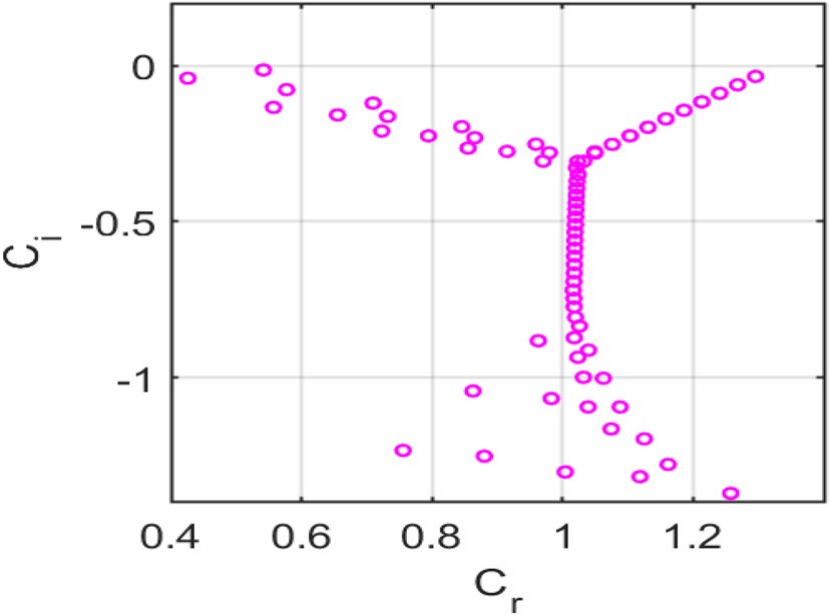
Eigenvalue spectrum for the general Navier-slip with slip lengths ${k}_{1}=0.1 and\,{k}_{2}=0.2$.

Figure 15 shows the eigenvalue spectrum for the classical no-slip flow, showing unstable modes $\left({\mathrm{c}}_{\mathrm{i}}>0\right),$ consistent with classical Tollmien-Schlichting instability. Figure 16 presents the eigenvalue spectrum for the symmetric Navier-slip case. Unlike the no-slip case, an eigenvalue lies above the neutral axis $\left({c}_{i}>0\right),$ indicating temporal instability due to a modified Tollmien–Schlichting mode. Symmetric slip reduces wall shear and shifts the spectrum toward higher phase speeds, destabilizing the flow. Figure 17 shows the eigenvalue spectrum for the asymmetric Navier-slip case, since all eigenvalues satisfy $\left({c}_{i}<0\right),$ the flow is stable under these conditions. Figure 18 shows the eigenvalue spectrum for the general Navier-slip case with unequal slip lengths, since all eigenvalues satisfy $\left({c}_{i}<0\right),$ The flow is stable under these conditions. All eigenvalues occur below the neutral axis $\left({c}_{i}<0\right),$ which indicates that the flow remains temporally stable. The absence of unstable Tollmien-Schlichting modes highlights that the critical Reynolds number has not yet been reached.

**Fig. 19:**
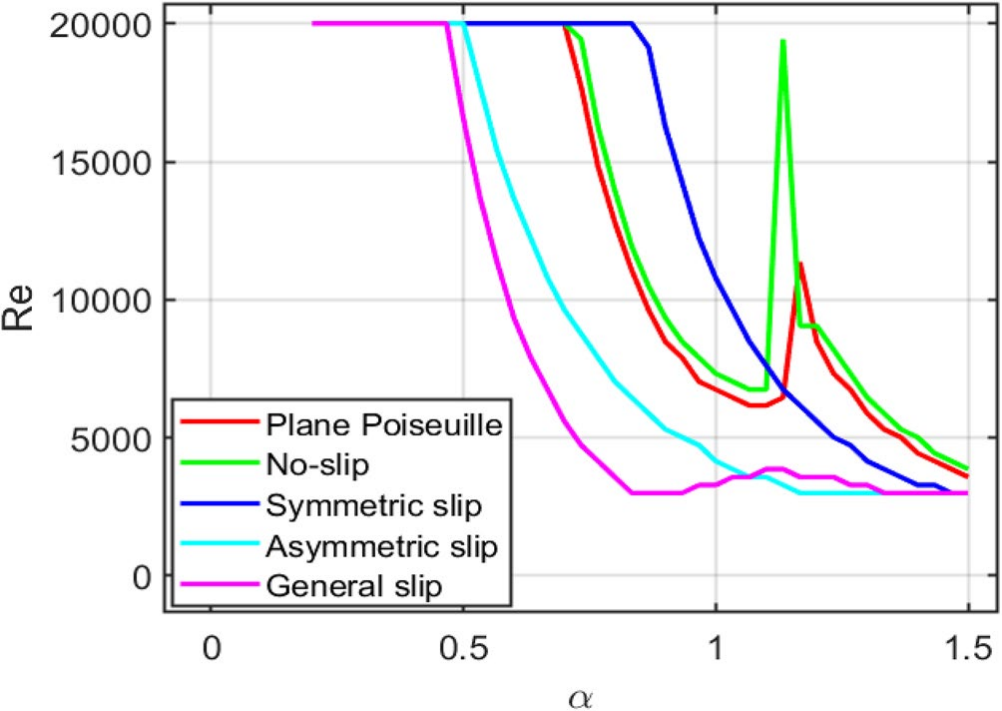
Combined neutral stability curves ($c={c}_{r}+i{c}_{i},\hspace{0.25em} {c}_{i}=0$) for $A=2$, $S=0.1$, $H=0.3$, $k=0.1$, ${k}_{1}=0.1$, ${k}_{2}=0.2$, computed using $N=120$ Chebyshev points with $\alpha \in \left[\mathrm{0.2,1.5}\right]$ and $Re\in [\mathrm{3000,20000}]$.

Figure 19 shows that the combined neutral stability curve represents the onset of linear instability for different flow configurations plotted on a single $\left(\alpha ,Re\right)$-plane. For each case, the curve is obtained by identifying the combinations of the Reynolds number $Re$ and streamwise wavenumber $\alpha$ for which the imaginary part of the complex phase speed vanishes, i.e., $c={c}_{r}+i{c}_{i},\hspace{0.25em} {c}_{i}=0.$

This condition separates stable disturbances $\left({c}_{i}<0\right)$ from unstable disturbances $\left({c}_{i}>0\right)$.

In the combined plot, each curve corresponds to a distinct base-flow configuration (plane Poiseuille flow, no-slip porous flow, symmetric Navier-slip, asymmetric Navier-slip, and general Navier-slip flow). The region above a given curve denotes unstable flow, whereas the region below the curve corresponds to stable flow. The minimum point on each neutral curve identifies the critical Reynolds number $R{e}_{c}$ and the associated critical wavenumber ${\alpha }_{c}$, marking the first appearance of instability.

By plotting all neutral curves together, the combined diagram provides a direct visual comparison of the stabilising or destabilising influence of wall slip and porous-medium/MHD effects. A shift of the neutral curve toward higher Reynolds numbers indicates a stabilising effect, while a shift toward lower Reynolds numbers signifies a destabilising influence. Differences in curve shape and location also reveal how symmetry or asymmetry in slip conditions modifies the preferred disturbance wavelength at the onset of instability.

The findings presented here are particularly significant for real-world systems that involve magnetohydrodynamic flow in porous channels with wall slip. The Lorentz force, which acts to reduce velocity, plays a critical role in the stabilising effect of the applied magnetic field. This effect alters and extends the onset of instability. Since magnetic fields are used to control flow behaviour in liquid-metal cooling systems, electromagnetic flow control, and various metallurgical processes, understanding this mechanism is essential. The effect of porous resistance indicates that reducing disturbance growth by varying the porous medium’s resistance enhances flow stability. In porous heat exchangers, packed-bed reactors, and filtration equipment, reducing flow instabilities can increase system stability and efficiency.

Additionally, wall sliding greatly alters the flow’s stability characteristics. By reducing wall shear stress and altering the velocity profile, improved slip can either stabilize or destabilize the flow, depending on the slip design. This behaviour is crucial in lubricant technologies, microfluidic systems, and flows across hydrophobic or developed surfaces where slip effects are significant.

## Conclusion

This work provides a thorough knowledge of the interactions between wall slip, magnetic damping, and porous resistance that affect the stability and energy behaviour of viscous channel flows. By employing the Chebyshev spectral collocation method to solve the modified Orr-Sommerfeld equation, this work demonstrates that altering boundary conditions can be a valuable strategy for promoting stable and energy-efficient fluid flow in engineered systems.

Symmetric wall slip reduces wall friction but can destabilise the flow, suggesting that careful design of surface textures and materials is crucial for achieving a balance between flow enhancement and stability. In contrast, asymmetric slip and porous resistance contribute to enhanced flow control and stability, providing pathways for improved performance in systems that rely on controlled transport processes, such as sustainable cooling, filtration, and clean energy conversion. The magnetic field exerts a stabilising influence by mitigating disturbances and reducing velocity gradients, contributing to improved operational reliability in magnetohydrodynamic and energy-harvesting applications.

The results show how durable, low-energy, and adaptable flow systems may be created by combining surface engineering with magnetic control. The sustainable technologies in microfluidics, energy-efficient cooling, renewable energy systems, and environmental engineering, where optimising flow behaviour promotes resource conservation and system stability, call for such understanding. This work thus supports the broader pursuit of sustainable industrial innovation, clean technology development, and the responsible management of energy and materials through advanced fluid-dynamic modelling.

The present analysis is confined to the evaluation of critical Reynolds numbers. In contrast, a comprehensive mapping of neutral stability curves and the determination of essential Hartmann or Darcy numbers are deferred to future studies.

Although the investigation provides valuable insights, it is subject to certain limitations. In particular, nonlinear effects are neglected, and the analysis is restricted to two-dimensional disturbances within the framework of linear stability theory. Future extensions of this work may incorporate nonlinear stability analysis, three-dimensional perturbations, and more complex fluid models, such as non-Newtonian or nanofluid flows. Furthermore, incorporating temperature-dependent properties, wall roughness, and time-dependent magnetic fields could yield more profound insights into practical flow control and real-world engineering applications.

## Data Availability

The data that support the findings of this study are available from the corresponding author [A.B.] upon reasonable request

## List of symbol

$U, V$: Dimensional velocity components in the x and y directions ($m\!\cdot\!{s}^{-1}$)
$X, Y$: Dimensional coordinates (streamwise, wall-normal) ($m$)
$u, v$: Dimensionless velocity components (dimensionless)
$x, y$: Dimensionless coordinates (dimensionless)
$$t$$: Time ($$s$$)
$a$: Channel half-width ($m$)
$${U}_{0}$$: Characteristic velocity ($$m\!\cdot\!{s}^{-1}$$)
$$p$$: Pressure ($$Pa$$)
$P$: Dimensionless pressure (dimensionless)
$$\rho$$: Fluid density ($$kg\!\cdot\!{m}^{-3}$$)
$$\mu$$: Dynamic viscosity ($$Pa\!\cdot\!{s}$$)
$$\nu$$: Kinematic viscosity ($${m}^{2}\!\cdot\!{s}^{-1}$$)
$K$: Permeability of the porous medium (${m}^{2}$)
$$\sigma$$: Electrical conductivity ($$S\!\cdot\!{m}^{-1}$$)
${B}_{0}$: Transverse magnetic field strength ($T (tesla)$)
$$k, {k}_{1}, {k}_{2}$$: Slip length ($$m$$)
$S$: Shape factor or porous resistance parameter(dimensionless)
$Da$: Darcy number (dimensionless)
$H$: Hartmann number (dimensionless)
$u^{\prime}, v^{\prime}$: Perturbation velocity components ($m\!\cdot{s}^{-1}$)
$\overline{u }(y)$: Base flow (mean velocity) profile ($m\!\cdot\!{s}^{-1}$)
$$\psi$$: Streamfunction ($${m}^{2}\!\cdot\!{s}^{-1}$$)
$\phi$: Wall-normal eigenfunction (dimensionless perturbation amplitude) (dimensionless)
$$\alpha$$: Wavenumber ($${m}^{-1}$$)
$c$: Complex wave speed ($m\!\cdot{s}^{-1}$)
U: Velocity vector $$\mathrm{U}=(U,V)$$ ($$m\!\cdot\!{s}^{-1}$$)
${\mathrm{B}}_{0}$: Applied magnetic field vector ($T$)

## References

[1] C. L. M. H. Navier Mémoire sur les lois du mouvement des fluids *Mém. l. Acad. R. Des. Sci. l. Inst. Fr.* 6 389 440 (1823).

[2] Lauga, E. & Cossu, C. A note on the stability of slip channel flows. *Phys. Fluid.*10.1063/1.2032267 (2005).

[3] He, Q. & Wang, X. The effect of the boundary slip on the stability of shear flow. *ZAMM. J. Appl. Math. Mech. Zeitschrift Für. Angew. Math. Und Mech*10.1002/zamm.200800020 (2008).

[4] Chai, C. & Song, B. Stability of slip channel flow revisited. *Phys. Fluid.*10.1063/1.5108804 (2019).

[5] Chen, K. & Song, B. Linear stability of slip pipe flow. *J. Fluid. Mech.* **910**, A35. 10.1017/jfm.2020.997 (2021).

[6] Lellep, M., Linkmann, M., Eckhardt, B. & Morozov, A. Purely elastic linear instabilities in parallel shear flows with free-slip boundary conditions. *J. Fluid. Mech.* **928**, R3. 10.1017/jfm.2021.840 (2021).

[7] Al-Zubaidi, A., Nazeer, M., Hussain, F. & Saleem, S. Numerical study of squeezing flow past a Riga plate with activation energy and chemical reactions: Effects of convective and second-order slip boundary conditions. *Waves Random Complex Med.* **35**(3), 5933–5946. 10.1080/17455030.2022.2072974 (2025).

[8] Javed, M. A., Akram, R., Nazeer, M. & Ghaffari, A. Heat transfer analysis of the non-Newtonian polymer in the calendering process with slip effects. *Int. J. Mod. Phys. B.*10.1142/S0217979224501054 (2024).

[9] Makinde, O. D. & Mhone, P. Y. On temporal stability analysis for hydromagnetic flow in a channel filled with a saturated porous medium. *Flow Turbul. Combust.* **83**(1), 21–32. 10.1007/s10494-008-9187-6 (2009).

[10] Smolentsev, S. MHD duct flows under hydrodynamic ‘slip’ condition. *Theor. Comput. Fluid Dyn.* **23**(6), 557–570. 10.1007/s00162-009-0108-7 (2009).

[11] Xu, Y.-J. et al. Electro-osmotic flow of biological fluid in divergent channel: Drug therapy in compressed capillaries. *Sci. Rep.* **11**(1), 23652. 10.1038/s41598-021-03087-0 (2021). 34880373 10.1038/s41598-021-03087-0 PMC8654908

[12] Kudenatti, R. B., Misbah, N.-E.- & Bharathi, M. C. Stability of hydromagnetic boundary layer flow of non-Newtonian power-law fluid flow over a moving wedge. *Eng. Comput.*10.1007/s00366-020-01094-9 (2022).

[13] Kudenatti, R. B., B, M. C. & Noor, E. M. Linear stability on transpiration effect of self-similar boundary layer flow for non-Newtonian fluids over a moving wedge. *Math. Comput. Simul.*10.1016/j.matcom.2024.11.016 (2025).

[14] Badday, A. J. & Harfash, A. J. Magnetohydrodynamic instability of fluid flow in a porous channel with slip boundary conditions. *Appl. Math. Comput.* **432**, 127363. 10.1016/j.amc.2022.127363 (2022).

[15] Thomas, C., Alveroğlu, B., Stephen, S. O., Al-Malki, M. A. S. & Hussain, Z. Effect of slip on the linear stability of the rotating disk boundary layer. *Phys. Fluid.*10.1063/5.0162147 (2023).

[16] Karmakar, S. & Shukla, P. Instability of a plane Poiseuille flow bounded between inhomogeneous anisotropic porous layers. *Therm. Sci. Eng. Prog.* **40**, 101758. 10.1016/j.tsep.2023.101758 (2023).

[17] Dabiri, J. O. & Leonard, A. Linear instability of viscous parallel shear flows: Revisiting the perturbation no-slip condition. *J. Fluid Mech.* **996**, A47. 10.1017/jfm.2024.806 (2024).

[18] Nazeer, M. et al. Poiseuille flow of Jeffrey fluid with variable transport properties in porous media under magnetic and radiative effects. *Dyn Atmos. Ocean.*10.1016/j.dynatmoce.2025.101599 (2025).

[19] Kumar, D. L. S., Geetha, D. L. & Basavaraj, M. S. Modal and non-modal linear stability analysis of plane channel flow through a Darcy-Brinkman porous medium with symmetric and asymmetric slippery walls. *Int. J. Non-Linear Mech.* **171**, 105015. 10.1016/j.ijnonlinmec.2025.105015 (2025).

[20] Nazeer, T., Hussain, M. & Anwar, F. Thermal analysis in transformer oil-based MHD two-phase flow of Prandtl fluid through inclined channel. *Comput. Method. Differ. Equ.*10.22034/cmde.2025.65293.2993 (2025).

[21] Hussain, F. et al. A note on the multiphase flow of third grade fluid with wall properties. *Waves. Random. Complex. Media.* **35**(3), 5947–5962. 10.1080/17455030.2022.2073400 (2025).

[22] Geetha, D. L. & Shivaraj, D. L. Stability of plane Poiseuille channel flow of a classical Newtonian fluid in the presence of uniform transverse magnetic field: Modal and non-modal approach. In *Innovative Solutions: A Systematic Approach Towards Sustainable Future* Edition 1 317–331 (BP International, 2025). 10.9734/bpi/mono/978-93-49238-47-3/CH33.

[23] Shivaraj, D. L., Geetha, D. L. & Basavaraj, M. S. Modal and nonmodal instabilities of a two-dimensional channel flow subject to wall slip and transverse magnetic field. *J. Fluid. Eng.*10.1115/1.4068134 (2025).

[24] Al‐Zubaidi, A., Nazeer, M., Anwar, T. & Saleem, S. Perturbation method for the motion of crystal solid particles in Couette–Poiseuille flow of non‐Newtonian fluid. *ZAMM Z. Angew. Math. Mech.*10.1002/zamm.70265 (2025).

[25] Nazeer, M. Development and theoretical analysis of slippery walls flow of third-grade fluid through the convergent symmetric channel. *Waves. Random. Complex. Media.* **35**(6), 11678–11697. 10.1080/17455030.2022.2123970 (2025).

[26] Lamesse, T. & Ibrahim, W. Modeling and optimization of unsteady MHD Casson-dusty nanofluid in a Darcy-Forchheimer variable porous medium via response surface methodology. *Result. Eng.* **28**, 107162. 10.1016/j.rineng.2025.107162 (2025).

[27] Lamesse, T. & Ibrahim, W. MHD flow and heat transfer of Carreau nanofluid with slip effects, and modified Fourier–Fick’s law heat–mass fluxes over a paraboloid surface in porous medium. *Result. Phys.* **72**, 108201. 10.1016/j.rinp.2025.108201 (2025).

[28] Lamesse, T. & Ibrahim, W. Magnetic Powell–Eyring nanofluid flow past a paraboloid surface with variable porosity. *Multiscale. Multidiscip. Model. Exp. Des.* **8**(8), 363. 10.1007/s41939-025-00955-4 (2025).

[29] Lamesse, T. & Ibrahim, W. Heat and mass transfer analysis of tangent hyperbolic nanofluid flow over a paraboloidal surface with quadratic mixed convection in porous medium. *Result. Eng.* **25**, 104459. 10.1016/j.rineng.2025.104459 (2025).

[30] Miles, J. Hydrodynamic stability (P. G. Drazin and W. H. Reid). *SIAM Rev.* **24**(3), 351–354. 10.1137/1024079 (1982).

[31] Squire, H. B. On the stability for three-dimensional disturbances of viscous fluid flow between parallel walls. *Proc. R. Soc. Lond. A Math. Phys. Sci.* **142**(847), 621–628. 10.1098/rspa.1933.0193 (1933).

[32] Drazin, P. G. & Reid, W. H. *Hydrodynamic Stability*10.1017/CBO9780511616938(Cambridge University Press, 2004).

[33] Schmid, P. J. & Henningson, D. S. Stability and Transition in Shear Flows. In *Applied Mathematical Sciences* 142 10.1007/978-1-4613-0185-1(New York, NY: Springer New York, 2001).

[34] Orszag, S. A. Accurate solution of the Orr–Sommerfeld stability equation. *J. Fluid. Mech.* **50**(4), 689–703. 10.1017/S0022112071002842 (1971).

